# Comparative transcriptome combined with morphophysiological analyses revealed the molecular mechanism underlying *Tetrahymena thermophila* predation-induced antiphage defense in *Aeromonas hydrophila*

**DOI:** 10.1080/21505594.2022.2127186

**Published:** 2022-09-24

**Authors:** Yuhao Dong, Jin Liu, Meng Nie, Dan Zhao, Hao Huang, Jinzhu Geng, Xihe Wan, Chengping Lu, Yongjie Liu

**Affiliations:** a Joint International Research Laboratory of Animal Health and Food Safety, College of Veterinary Medicine, Nanjing Agricultural University, Nanjing, China; b Hubei Key Laboratory of Animal Nutrition and Feed Science, Wuhan Polytechnic University, Wuhan, China; c Institute of Oceanology and Marine Fisheries, Nantong, China

**Keywords:** *Aeromonas hydrophila*, *tetrahymena thermophila*, predation, *flhf*, phage resistance

## Abstract

Protozoan predation has been demonstrated to be a strong driving force for bacterial defence strategies in the environment. Our previous study demonstrated that *Aeromonas hydrophila* NJ-35, which evolved small-colony variants (SCVs), displayed various adaptive traits in response to *Tetrahymena thermophila* predation, such as enhanced phage resistance. However, the evolutionary mechanisms are largely unknown. In this study, we performed a genome- and transcriptome-wide analysis of the SCV1, representing one strain of the SCVs, for identification of the genes of mutation and altered expression underlying this phage resistance phenotype. Our study demonstrated that phage resistance caused by *T. thermophila* predation was due to the downregulation of a flagellar biosynthesis regulator, *flhF*, in SCV1. Interestingly, we confirmed that phage resistance in SCV1 was not straightforwardly attributable to the absence of flagella but to FlhF-mediated secretion of extracellular protein that hinders phage adsorption. This finding improves our understanding of the mechanisms by which *A. hydrophila* lowers the susceptibility to phage infection under predation pressure, and highlights an important contribution of bacterium–protozoan interactions in driving the adaptive evolution of pathogens in complex environments.

## Introduction

The rapidly changing environment is increasingly recognized as a strong driving force for the evolution of microbial defence strategies [1]. *Aeromonas hydrophila* is a ubiquitous motile bacterium in aquatic environments that causes gastroenteritis and skin infections in humans and motile aeromonad septicaemia (MAS) in freshwater fish [2]. It employs several defence strategies to survive and adapt to the complex environment, including biofilm formation, production of toxic secondary metabolites and nutrient acquisition in the host [3,4]. Moreover, bacteria in the environment are often threatened by predators such as protozoa and nematodes, which are thought to be considerable factors that can trigger adaptive evolution in bacteria [5,6].

Protists are single-celled eukaryotes that are ubiquitous in almost all environments. Protozoa have been described as the “Trojan horses of the microbial world” since they have the ability to promote the survival of pathogenic bacteria in various environmental conditions [7]. Protists take up bacterial prey into phagosomes that become acidified and filled with enzymes, resulting in digestion [8]. However, some bacteria, such as *Salmonella enterica* serovar Typhimurium [9], *Vibrio cholerae* [10], *Campylobacter jejuni* [11] and *A. hydrophila* [12], can prevent the fusion of lysosomes and phagosomes, thereby avoiding digestion [13]. Notably, bacteria released from food vacuoles become more capable to survive in harsh environments. For example, coculture of *S*. Typhimurium with *T. thermophila* showed more resistance to acidic environments [9]; *Acanthamoeba castellanii* predation enhanced the survival of *Legionella pneumophila* under starvation conditions and may remain viable for at least six months in poor medium [14].

Although the mechanisms by which bacteria have evolved to respond to protistan predation are not fully understood, the defensive strategies, including size reduction, microcolony, and biofilm formation, alterations in motility, and surface masking, provide strong support for enhanced survival [15–17]. Our previous study indicated that *A. hydrophila* exhibited decreased susceptibility to phage infection after exposure to *T. thermophila* predation, although the specific mechanism is elusive [12]. Phages are viruses that can attack bacteria, thereby controlling the bacterial population. Phage resistance has been considered to be a crucial survival phenotype in the face of environmental change [18]. To defend against phage infection, bacteria have evolved a battery of resistance mechanisms, such as escaping phage adsorption, preventing phage DNA injection and digesting phage nucleic acids [19]. However, the role of predation-induced activation of genes in controlling resistance to phage is largely unexplored. Here, high-throughput whole genome sequencing and RNA sequencing (RNA-Seq) are performed to assess the genetic variations and transcriptomic changes, and identify the key genes involved in antiphage defence in *A. hydrophila* under the pressure of *T. thermophila* predation. A flagellar regulator gene, *flhF*, was found to be significantly downregulated, which led to a remarkable increase in protein secretion and thus prevented phage adsorption by providing a physical barrier between phage and its receptors. The findings of this study provide novel insight into the evolution of bacterial defence strategies.

## Materials and methods

### Strains, cell lines, and media

The bacterial strains and plasmids used in this study are listed in Table S1. *A. hydrophila* NJ-35 and its derivative non-*Tetrahymena-*exposed strain B1 (conventionally cultured without *T. thermophila*) and *Tetrahymena-*exposed strain SCV1 (a small colony variant (SCV) strain after cocultured with *T. thermophila*) were maintained in Luria-Bertani (LB) medium at 28°C, as previously described [12]. *Escherichia coli* SM10 was conventionally cultured in LB medium at 37°C [12]. When necessary, chloramphenicol (Cm) (Sigma Louis, MO, USA), kanamycin (Kan) (Sigma), or ampicillin (Amp) (Sigma) were added to the medium. *T. thermophila* SB210 (accession number GCA_000261185.1) was obtained from Dr. Miao Wei, Institute of Hydrobiology, China Academy of Sciences, and cultured in SPP medium (2% protease peptone, 0.1% yeast extract, 0.2% glucose, 0.003% EDTA-Fe) at 28°C [12]. The lytic bacteriophage G65, belonging to the family *Myoviridae*, was isolated from a contaminated river in Nanjing, China, in 2014 [20].

### DNA isolation, resequencing, and data analysis

*A. hydrophila* strains were cultured overnight at 28°C in LB medium. Genomic DNA was extracted using the QIAGEN Genomictip 100/G (Qiagen, CA, USA). The Qubit 4.0 Fluorometer for double-strand-DNA high-sensitivity assay kit (Thermo Fisher Scientific, MA, USA) was used to quantified the concentrations of DNA. Whole-genome sequencing was performed on the PacBio RS II platform (Pacific Biosciences, CA, USA). The sequencing reads were assembled using Falcon version 0.7.0 and FALCON-Unzip. The presence of single-nucleotide polymorphisms (SNPs) and other genomic variations were analysed using CLC Genomic Workbench 5.5.1 probalistic variant detection software.

### RNA-Seq and data analysis

Total RNA from strains B1 and SCV1 was isolated using a mirVana miRNA Isolation Kit (Ambion, TX, USA). Then, the integrity and quality of RNA were assessed using an Agilent 2100 Bioanalyzer (Agilent Technologies, CA, USA). Then, RNA-seq libraries were prepared using the Illumina sequencing platform (HiSeq^TM^ 2500) by OE Biotech (Shanghai, China). Transcriptome reads were mapped to the reference genome of *A. hydrophila* NJ-35 using TopHat2 software. DESeq1.18.0 was used to perform differential expression analysis of two strains (three biological replicates per strain). Genes with a *P* value ≤0.05 and a fold change in expression between two samples of more than 2.0 were designated as differentially expressed.

### Quantitative reverse transcription-PCR (qRT-PCR)

Total RNA was extracted from a logarithmic-phase bacterial culture using an E.Z.N.A. Bacterial RNA Kit (Omega, GA, USA). cDNA synthesis was conducted using HiScript II QRT Supermix (Vazyme, Nanjing, China). The mRNA transcription levels were examined using a One Step qRT-PCR SYBR Green Kit (Vazyme, Nanjing, China) in a StepOnePlus™ Real-Time PCR System (Applied Biosystems, MA, USA). The fold-change in mRNA expression value was calculated using the 2^−ΔΔCT^ method and normalized against the expression level of the housekeeping gene *recA*. The primers used are listed in Table S2. The assay was performed in three independent biological experiments.

### Construction of deletion mutants and complementation

The gene mutant was constructed by homologous recombination [21]. The left and right arms flanking the targeted gene were amplified using the genome of the *A. hydrophila* B1 strain as a template. Then, the fusion fragments were cloned into the suicide plasmid pYAK1 (chloramphenicol resistance, Cm^r^) with the restriction enzyme *Bam*HI [4,22]. The recombinant plasmid was conjugated into the *A. hydrophila* B1 strain (ampicillin resistance, Amp^r^). Colonies that had undergone allelic exchange were selected on LB agar plates with Amp and Cm. The positive colonies were inoculated into LB broth containing 20% sucrose. The suspected positive colonies were screened by PCR and verified by sequencing.

The corresponding complemented strain of the mutant was constructed with the shuttle plasmid pMMB207 [23]. The complete open reading frame (ORF) of the target gene was isolated by PCR amplification and inserted into pMMB207. Then, the recombinant plasmid was transformed into the mutant by conjugation. The complemented mutant was selected on LB agar with Amp and Cm, and further verified by PCR. In addition, the recombinant plasmid was transformed into SCV1 to generate the overexpression strain. The primers used are listed in Table S2.

### Dynamic growth curve of bacteriophage

Overnight-cultured *A. hydrophila* was adjusted to a concentration of 5 × 10^8^ CFU/mL and transferred into 20 mL of LB media at a dilution of 1:100. Bacteriophage G65 was applied to the bacterial culture to yield a final concentration of 5 × 10^4^ PFU/mL, and the cocultures were incubated at 28°C for 24 h with 180 rpm shaking. Phage cultured alone served as a control. Phage enumeration was determined at a regular interval of 2 h between this 24-h incubation period by counting the plaques using a double-layer plaque assay.

To determine whether bacterial surface proteins or polysaccharides were involved in phage-bacterial host recognition, *A. hydrophila* strains were cultured under the same conditions as shown above except that either 0.1 mg/mL proteinase K (Dingguo, Nanjing, China) or 2.14 mg/mL sodium *meta*-periodate (NaIO_4_; Dingguo) was added to the medium prior to coculture with phage G65. The samples were treated with proteinase K or NaIO_4_ at 37°C for 2 h. The assay was performed in three independent biological experiments.

### Determination of phage adsorption kinetics

*A. hydrophila* strains grown to logarithmic phase were adjusted to an OD_600_ of 0.01 (5.0 × 10^6^ CFU/mL). Nine millilitres of the bacterial suspension was added to 1 mL of phage G65 (5.0 × 10^5^ PFU/mL). Phages mixed with fresh LB medium served as a negative control. At 2.5 min intervals, 50 μL of each coculture was added to 950 μL of LB medium precooled on ice. The mixtures were centrifuged at 10,000×*g* for 10 min. The titres of the free phages in the supernatants were determined by a double-layer plaque assay.

To determine whether surface proteins or polysaccharides were involved in phage adsorption, the bacteria were treated with proteinase K (0.1 mg/mL) or NaIO_4_ (2.14 mg/mL) for 2 h, and then washed using LB medium before coculture with phage G65. The assay was performed in three independent biological experiments.

### Motility assay

*A. hydrophila* strains were cultured to logarithmic phase and adjusted to an OD_600_ of 1.0. 0.3% LB agar plates were stabbed with 1 µl of each culture. Motility was assessed by measuring diameters of the outermost rings after incubating for 48 h at 28°C. The assay was performed in three independent biological experiments.

### Western blot analysis

Bacteria were grown to an OD_600_ of 2.0, and the number of colony-forming units (CFUs) was determined before pelleting the cells. The cells were resuspended in SDS-PAGE loading buffer to obtain equal cell numbers per microlitre. The supernatant of each strain was filtered using a 0.22-μm Millipore filter and subsequently treated with SDS-PAGE loading buffer. Proteins were separated using 12.5% SDS-PAGE and electrophoretically transferred to nitrocellulose filter membranes (NC membranes). The membranes were incubated with a primary antibody against the protein of interest, followed by horseradish peroxidase (HRP)-conjugated goat anti-rabbit IgG (1:5000; Linc-Bio Science, Shanghai, China) for 2 h. Detection of GroEL was performed as a loading control for both whole-cell and supernatant proteins. The blots were visualized using an Omni-ECL Kit (EpiZyme, Shanghai, China) and a ChemiDoc^TM^ Touch imaging system (Bio-Rad, CA, USA). The assay was performed in three independent biological experiments.

### Transmission electron microscopy (TEM)

*A. hydrophila* colonies grown for 20 h were thoroughly suspended in sterile water. A 15-μL aliquot of bacterial solution was placed on a 200-mesh Formvar-coated copper microscopy grid. The grid was baked for 5 to 10 s under incandescent light. The bacteria were then negatively stained with 15 µL of 2% (wt/vol) phosphotungstic acid for one minute and dried. All samples were examined on a Hitachi 600 transmission electron microscope (Hitachi, Tokyo, Japan).

### Gel electrophoresis of extracellular proteins

Bacterial cells were grown to the logarithmic phase for 8 h at 28°C. Cultures were adjusted to an OD_600_ of 2.0. Culture supernatants were harvested by centrifugation and filtered through a 0.22-μm filter. Protein concentration was determined by the bicinchoninic acid (BCA) assay. Proteins contained in supernatants were precipitated by treatment with 10% trichloroacetic acid (TCA, Sigma-Aldrich, MO, USA). After washing with acetone, the precipitates were dissolved in SDS sample buffer. Protein samples of equal volume were boiled for 10 min and subjected to 12.5% SDS-PAGE. The gels were visualized by silver staining. The assay was performed in three independent biological experiments.

### Measurement of haemolytic activities

*A. hydrophila* strains were cultured for 18 h at 28℃ and adjusted to an OD_600_ of 2.0. The supernatants were harvested by centrifugation and filtration. Next, 100 μL of sterilized saline was dispensed into each well of a 96-well polystyrene plate. An aliquot of the supernatant was added to the first well, and 2-fold dilutions were performed. Then, 100 μL of 2% sheep red blood cells (RBCs; Dingguo) was added to each well. RBCs (2%) mixed with an equal volume of sterilized saline or water served as the negative or positive control, respectively. After incubation for 1 h at 37°C, the plate was placed at 4°C overnight, and centrifugation was then performed to precipitate the unlysed cells at 4°C and at 800×*g*. One hundred microlitres of each supernatant was transferred to another 96-well plate, the optical density was measured at OD_540_. The assay was performed in three independent biological experiments.

### Antibacterial competition assay

The antibacterial competition assay was performed by detecting the surviving *E. coli* cells after coculturing with *A. hydrophila*. The *A. hydrophila* strains and *E. coli* BL21 with kanamycin resistance (Kan^r^) grown to logarithmic-phase were concentrated to 5 × 10^9^ CFU/mL and then mixed at a ratio of 1:1. Each mixture of 25 μL was added onto a nylon filter, which facilitates close contact between bacteria, on an LB agar plate. *E. coli* BL21 cells mixed with fresh LB medium served as the control. After incubation for 3 h at 28°C, the bacteria were collected and suspended in LB medium with a subsequent 10-fold serial dilution. The anti-*E. coli* competition ability of *A. hydrophila* strains was shown as the CFU count of viable *E. coli* cells after *A. hydrophila* challenge. The survival of *E. coli* at each dilution was evaluated using the viable cell count on LB agar plates containing Kan (50 μg/mL). The assay was performed in three independent biological experiments.

### Construction of point mutation

The fragment containing the point mutation was synthesized and cloned into the pYAK1 suicide plasmid. The recombinant plasmid was transferred into *E. coli* SM10 competent cells. The donor strain *E. coli* SM10-pYAK1 (Cm resistant, Cm^r^) and the recipient strain B1 (Amp resistant, Amp^r^) grown to log phase in LB broth without antibiotics were mixed at a ratio of 2:1 (vol/vol), and the cell pellets were then spotted onto a nylon filter overlaid on LB plates without antibiotics and incubated for 12 h at 28°C. The bacteria were harvested from the filter and grown on LB agar plates containing 100 µg/mL Amp and 34 µg/mL Cm. Then, the positive colonies (Amp^r^ and Cm^r^) were picked and inoculated into LB broth containing 20% sucrose to induce a second crossover event. The point mutant was verified by sequencing the mutated region of the target gene. The schematic diagram of the construction of the point mutant was shown in Figure S1.

### Statistical analyses

Data were collected and analysed using GraphPad Prism version 5 software. The phage resistance, motility, haemolytic activity, and antibacterial competition capability of the *A. hydrophila* parental strain and the derived mutants were analysed using *t*-tests. *P*-values <0.05 were considered statistically significant.

## Results

### Identification of acquired mutations in SCV1 by whole genome sequencing

**Figure 1.**
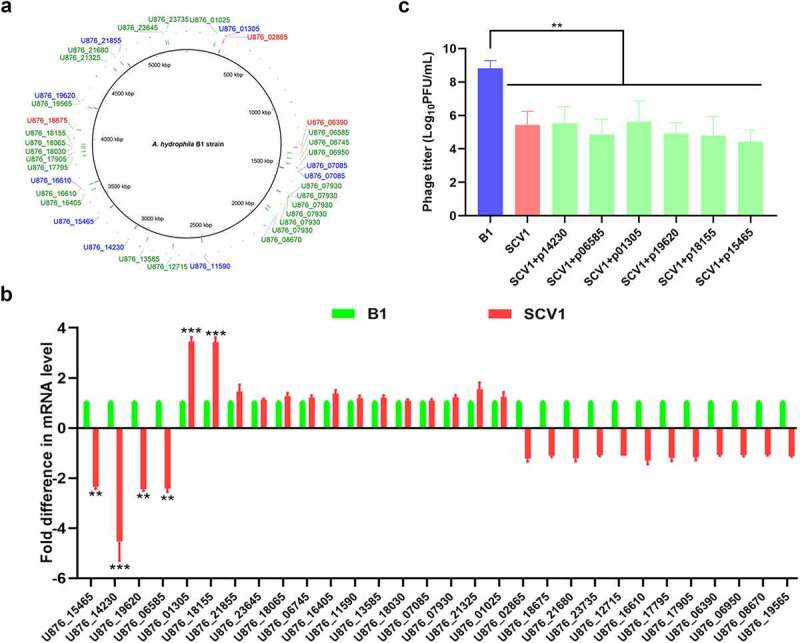
Mutations in SCV1 is not involved in phage resistance. (a) Mutations identified in the SCV1 strain compared to the B1 strain by whole-genome sequencing. The sequencing datasets of SCV1 were analysed using the B1 genome as a reference. 3 deletions, 26 insertions and 9 SNPs in the SCV1 were identified by mapping to the genome of B1 strain. The position of mutant bases was shown in the genome of reference strain B1. The deletions were in red font; the insertions were in green font; the SNPs were in blue font. Image is generated using BRIG (http://brig.sourceforge.net). (b) the relative transcription levels of the 30 mutated genes in the SCV1 strain compared with the B1 strain. (c) the titre of phage G65 cocultured with B1, SCV1 or its complementation strain with a single gene (U876_15465, U876_14230, U876_19620, U876_06585, U876_01305 or U876_18155) amplified from the B1 genome. The values are the mean ± sd from three biological replicates. ** *P* <0.01 or ***P <0.001.

To elucidate the genetic changes associated with the evolution of SCV1 under *T. thermophila* predation pressure, whole genome sequencing of the SCV1 and B1 strains was performed using the PacBio RS II platform. A total of 38 site variations occurring in 30 open reading frames (ORFs), including 3 deletions, 26 insertions, and 9 SNPs, were identified in the SCV1 strain compared with the B1 strain (Figure 1(a)). All nine SNPs in coding regions were nonsynonymous. The above gene alterations were confirmed by PCR amplification, followed by sequencing of the corresponding PCR products. Among the 30 mutated genes, 13 were annotated as hypothetical proteins of unknown function. The remaining 17 genes could be functionally characterized into four categories (Table S3): 4 genes were involved in transport, including ion transport and drug efflux; 5 genes were involved in metabolism/stress response, including enzymes involved in the TCA cycle and protein modification; 6 genes were involved in cell division/replication, including genes associated with replication and genetic regulation; and 2 transcriptional regulators were identified, including an AraC family transcriptional regulator, which has been reported to be involved in a variety of cellular processes from carbon metabolism to stress responses and the regulation of virulence [24]. qRT-PCR was performed to determine the transcriptional levels of the 30 mutated genes in the SCV1 strain. As shown in Figure 1(b), four genes (U876_15465, U876_14230, U876_19620, U876_06585) were downregulated and two (U876_01305, U876_18155) upregulated in the SCV1 strain, as compared with the B1 strain. To determine whether the mutation in these six genes caused an enhanced phage resistance phenotype observed in SCV1, we amplified the corresponding genes in the B1 strain and complemented them into SCV1, respectively, using shuttle plasmid pMMB207. The result showed that the biomass of phage G65 cocultured with SCV1 was lower than that cocultured with the B1 strain. However, none of the complemented strains (SCV1 + p15465, SCV1 + p14230, SCV1 + p19620, SCV1 + p06585, SCV1+p01305, and SCV1 + p18155) exhibited significant alterations in phage sensitivity compared to the SCV1 strain (Figure 1(c)). In a similar manner, we complemented the other 24 genes, whose transcription levels have not been unchanged, in the SCV1 background. As expected, the complemented strains did not restore phage sensitivity (Figure S2).

### A. hydrophila *genes preferentially expressed under* T. thermophila *predation pressure were identified*

**Figure 2.**
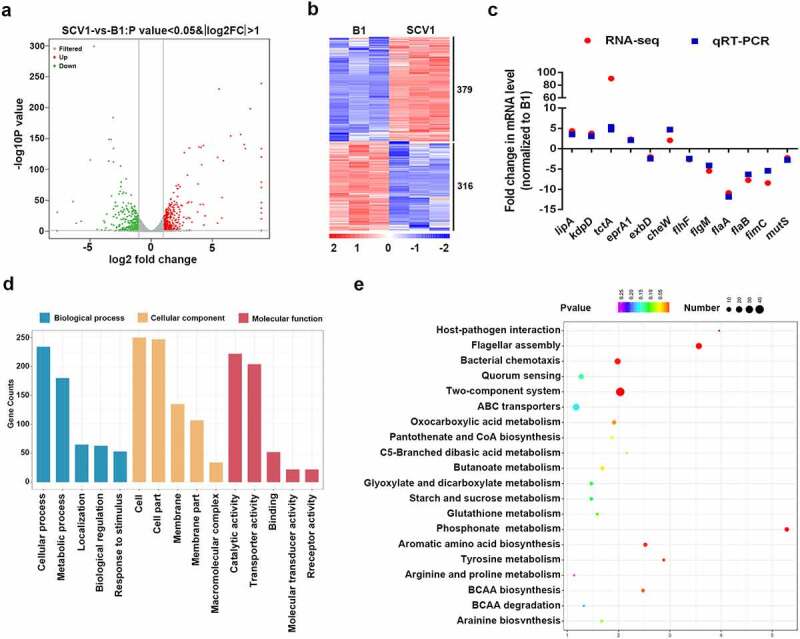
Comparative transcriptomics analysis. (a) Volcano plot of differentially expressed genes between SCV1 (*Tetrahymena*-exposed) and B1 (non-*Tetrahymena*-exposed). (b) Clustering heatmap of DEGs between the B1 and SCV1 strains. (c) qRT-PCR validation of the transcription of selected DEGs. The data indicate the fold change in the expression of genes in SCV1 compared with B1. (d) GO enrichment analysis. (e) KEGG pathway analysis.

To better understand the molecular mechanism of *A. hydrophila* evolution in response to *Tetrahymena* predation, we explored the differentially expressed genes (DEGs) between the SCV1 and B1 strains by transcriptomic analysis. A total of 695 DEGs (379 and 316 genes with significant down- or upregulation, respectively) were identified in SCV1 compared with B1 strain (Figure 2(a,b), Table S4). Most of these genes were related to basic physiologic and metabolic functions. To evaluate the reliability of RNA-Seq, qRT-PCR was performed on 12 selected genes of interest using RNA samples. All 12 genes showed strong agreement and were highly correlated in the RNA-Seq and qPCR analyses (Figure 2(c)). To facilitate a closer comparison of DEGs between the SCV1 and B1 strains, a pairwise comparison was conducted using GO assignments. A total of 695 DEGs were enriched in 62 GO terms. The highly enriched GO terms were mainly associated with the biological process of cellular process, metabolic process and localization, and the molecular functions were mainly associated with catalytic activity, transporter activity, and binding. The top 15 enriched GO terms are shown in Figure 2(d). To further elucidate the biological functions and interactions of the gene products, significantly affected pathway analysis was performed using the KEGG pathway database. The DEGs were mapped to 27 pathways. The main metabolic pathways of unique sequences were flagellar assembly, signal transduction, and metabolism. The top 20 enriched KEGG pathways are shown in Figure 2(e).

### *Downregulation of* flhF *is involved in cell aggregation and phage resistance in SCV1*

**Figure 3.**
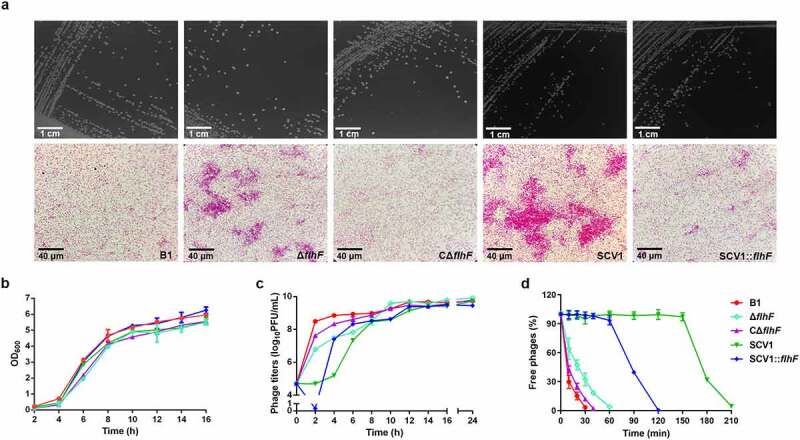
Downregulation of *flhF* is involved in cell aggregation and phage resistance in SCV1 (a) Colony (scale bar = 1 cm) and microscopic morphology (scale bar = 40 μm) of B1 and its derivative *flhF* mutant (Δ*flhF*) and the complemented strain (CΔ*flhF*), as well as SCV1 and its *flhF*-overexpressing strain (SCV1:*flhF*). (b) Growth curves of B1, Δ*flhF*, CΔ*flhF*, SCV1, and SCV1:*flhF*. (c) the titre of phage G65 cocultured with B1, Δ*flhF*, CΔ*flhF*, SCV1, and SCV1:*flhF*. (d) Adsorption kinetics of phage G65 to the indicated strains. The adsorption curve was drawn with time as the abscissa and the percentage of unadsorbed free phages as the ordinate. The data are presented as the mean ± SD of three independent experiments, with each experiment consisting of three replicates.

Based on the transcriptomic data, we identified that the *flhF* gene, encoding an SRP-type GTPase, was downregulated in the SCV1 strain. FlhF is a flagellar biogenesis regulator that has been reported to be involved in phage resistance in *Campylobacter jejuni* [25]. To determine the effect of the *flhF* gene on microscopic morphology and phage infection of *A. hydrophila*, the Δ*flhF* mutant was constructed in the B1 background. The deletion of *flhF* did not change the colony morphologies when grown on LB plates (Figure 3(a)). Under the light microscope, all the bacterial cells were of normal size and rod shape, but the Δ*flhF* and SCV1 strains displayed aggregations of large cell clusters compared with the B1 and CΔ*flhF* strains (Figure 3(a)). Notably, the overexpression of *flhF* in SCV1 resulted in decreased aggregation, indicating that downregulation of *flhF* in SCV1 is responsible for aggregation. Before examining phage infection, we investigated the growth ability of different bacterial strains. None of the strains showed any significant differences in bacterial growth when cultured in LB medium for 16 h (Figure 3(b)). *A. hydrophila* cultures at the exponential growth phase were infected with phage G65, and the phage titres were then measured at 2 h intervals. As shown in Figure 3(c), the biomass of phage G65 cocultured with Δ*flhF* was lower than that cultured with the B1 strain, while the overexpression of *flhF* in SCV1 (SCV1:*flhF*) yielded larger quantities of phage biomass than SCV1 from 4 to 8 h after treatment with phage G65. Moreover, the percentage of free phages was higher after coculture with Δ*flhF* than with B1 strain, 32.29% (Δ*flhF*) *vs* 3.35% (B1) at 30 min (Figure 3(d)). Complementation of *flhF* almost completely restored the B1 phenotype. In addition, phage adsorption to SCV1:*flhF* could be completed within 120 min, while phage adsorption to SCV1 required 210 min (Figure 3(d)), indicating that SCV1:*flhF* was more sensitive to phage infection than SCV1.

### Phage resistance of SCV1 is independent of the impaired flagellar production

**Figure 4.**
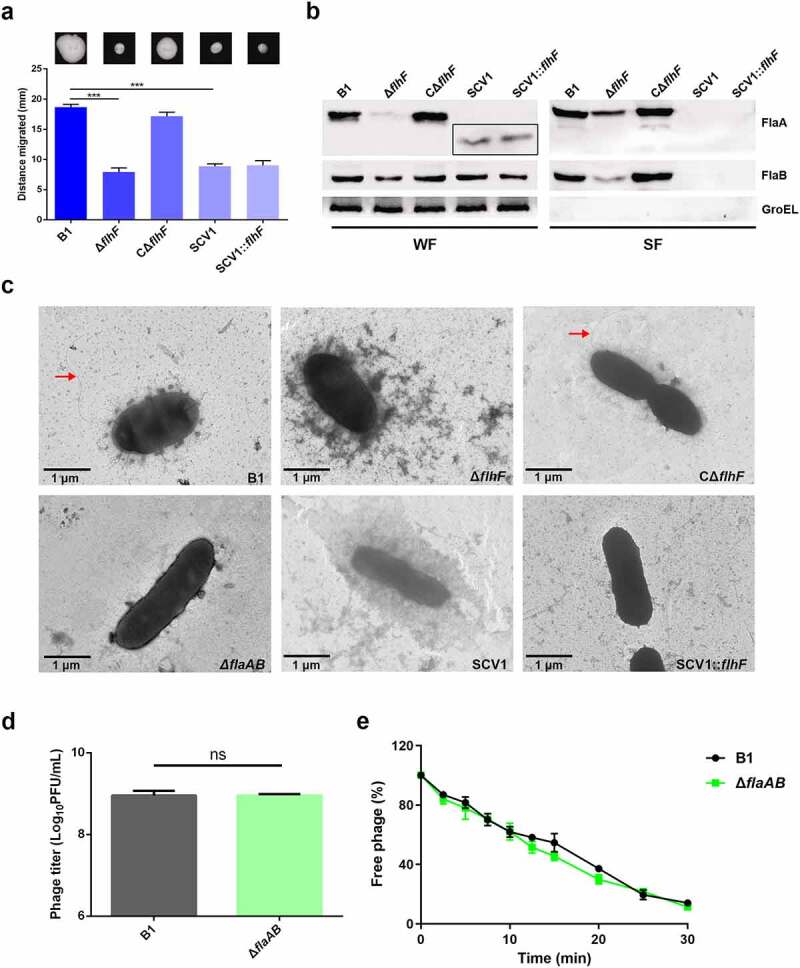
Phage resistance of SCV1 is independent of the impaired flagellar production. (a) Motility of B1, Δ*flhF*, CΔ*flhF*, SCV1, and SCV1:*flhF*. Motility was observed after culturing at 28°C for 48 h on 0.3% LB agar plates. Migration diameter was measured to assess motility. (b) the expression and secretion of flagellins FlaA and FlaB analysed by western blotting. Polyclonal anti-FlaA and anti-FlaB antibodies were used to measure the production of FlaA and FlaB, respectively. The whole-cell fractions (WF) and supernatant fractions (SF) were harvested, respectively, when the indicated strains were grown to logarithmic phase in LB broth. The samples were probed with anti-GroEL antibody as a cell lysis control for SF and a loading control for WF. (c) TEM images of the indicated strains. Scale bar = 1 μm. The red arrow indicates the flagellum. (d) the titre of phage G65 coculture with the B1 and Δ*flaAB* strains. The concentration of phages was determined using standard double agar overlay plaque assay. Phage titre as indicated by log10 PFU per mL. (e) Adsorption kinetics of G65 to B1 and Δ*flaAB* strains. The adsorption curve was drawn with time as the abscissa and the percentage of unadsorbed free phage as the ordinate. The data are presented as the mean ± SD of three independent experiments, with each experiment consisting of three replicates. “ns” signifies not statistically significant.

It has been reported that flagella are required for phage resistance in some bacteria, such as, *Agrobacterium* [26] and *Salmonella* [27]. In this study, we found that both Δ*flhF* and SCV1 showed decreased motility compared with the B1 strain (Figure 4(a)). Then, we evaluated the production of two repeating flagellin subunits, FlaA and FlaB, in Δ*flhF* and SCV1. The deletion of *flhF* resulted in markedly reduced FlaA/B expression and secretion, while SCV1 displayed low levels of FlaA/B expression and completely undetectable secretion of FlaA/B (Figure 4(b)). Also of note is that FlaA in the cytoplasm of SCV1 exhibited a significantly smaller molecular mass than the B1 and Δ*flhF* strains, as demonstrated by migration on the SDS-PAGE gel (Figure 4(b)). As expected, TEM images revealed that both Δ*flhF* and SCV1 completely lacked flagellation (Figure 4(c)). These findings led us to speculate that the phage resistance of Δ*flhF* and SCV1 may be due to the absence of flagella. To validate this hypothesis, however, we needed to first determine whether flagella are the receptor of phage G65. We inactivated the *flaA* and *flaB* genes, which encode the two structural proteins of the polar flagellum, in the B1 background. As shown in Figure 4(d), there was no difference in phage titre between the B1 and Δ*flaAB* strains. Similarly, the phage adsorption rate to the Δ*flaAB* mutant was the same as that to the B1 strain (Figure 4(e)). In addition, overexpression of the *flhF* gene in SCV1 (SCV1:*flhF*) did not restore the motility (Figure 4(a)) or flagellum structure (Figure 4(c)) but exhibited a higher sensitivity to phage infection than SCV1, as shown in Figure 3(c), further supporting that phage resistance in SCV1 is not associated with *flhF*-mediated regulation of flagella.

### Phage resistance of SCV1 is associated with excessive proteins on the bacterial cell surface

**Figure 5.**
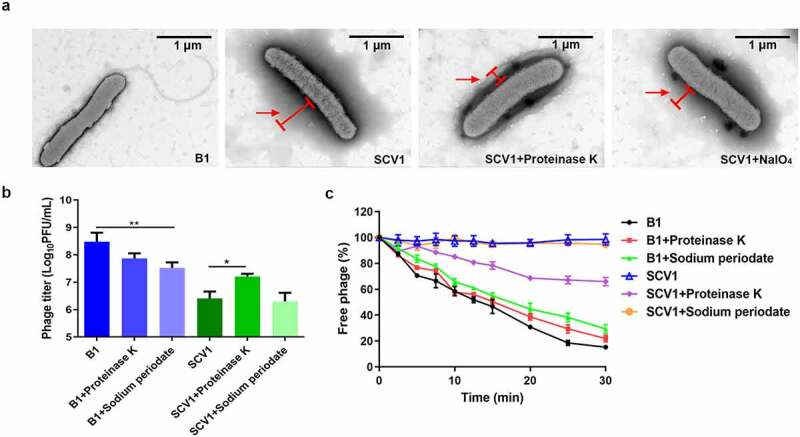
Protein components of the extracellular matrix hinder the adsorption of phage G65 to SCV1. (a) TEM images of the B1 strain and the SCV1 strain with or without proteinase K or NaIO_4_ treatment. Scale bar = 1 μm. The SCV1 strain was treated with proteinase K (0.1 mg/ml) or NaIO_4_ (2.14 mg/ml) for 2 h after growth in LB medium to logarithmic phase and then observed by TEM. The red arrow indicates the extracellular matrix. (b) the titre of phage G65 after incubation with the SCV1 strain with or without proteinase K or NaIO_4_ treatment. Proteinase K (0.1 mg/ml) or NaIO_4_ (2.14 mg/ml) was added 2 hours prior to coculture with phage G65. The concentration of phages was determined using standard double agar overlay plaque assay. Phage titre as indicated by log10 PFU per mL. The B1 strain with the same treatments as a control. (c) Adsorption kinetics of G65 to the B1 and SCV1 strains after treatment with proteinase K or NaIO_4_. the adsorption curve was drawn with time as the abscissa and the percentage of unadsorbed free phage as the ordinate. * *P* <0.05, ** *P* <0.01.

Based on TEM observations, we found that the surface of SCV1 was covered by a dense surface-associated coating, whereas no similar surface layer was present on the surface of B1 (Figure 5(a)). To investigate the possible component of the coating, we treated bacterial cultures with proteinase K or NaIO_4_. After treatment with proteinase K, the surface covering was significantly reduced in SCV1, while NaIO_4_ treatment showed no remarkable effect on the cell surface structure (Figure 5(a)). In addition, treatment with proteinase K caused a significant increase in the phage titre (Figure 5(b)) and phage adsorption rate (Figure 5(c)) compared with those of the control (without proteinase K). Similarly, treatment with NaIO_4_ did not affect phage titre or phage adsorption to SCV1. These data suggested that the protein components on the SCV1 surface may be an important factor influencing phage adsorption.

### *Downregulation of* flhF *in SCV1 is responsible for excessive extracellular proteins*

**Figure 6.**
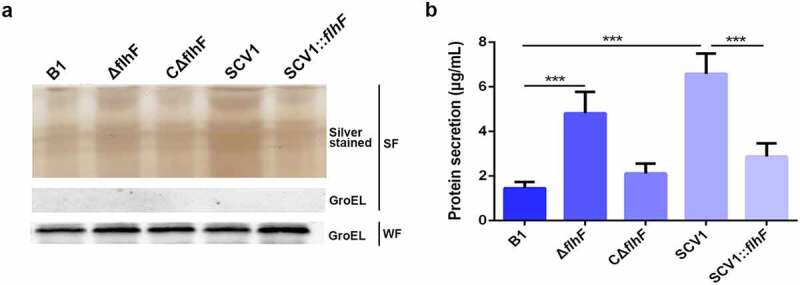
Downregulation of *flhF* in SCV1 is responsible for excessive extracellular proteins. (a) SDS-PAGE analysis of the culture supernatants of B1, Δ*flhF*, CΔ*flhF*, SCV1 and SCV1:*flhF*. Culture supernatants collected from logarithmic-phase cultures normalized at the same OD_600_ were separated by SDS-PAGE and silver stained. The samples were probed with α-GroEL as a cell lysis control for secreted fractions and a loading control for whole-cell fractions. WF: whole-cell fractions; SF: secreted fractions. (b) the levels of extracellular protein of the indicated strains. The supernatant fractions were harvested when the *A. hydrophila* strains were grown for 6 hours in LB broth. Protein secretion was measured by the BCA assay.

To characterize the effect of *flhF* downregulation on protein secretion by SCV1, the culture supernatants of *A. hydrophila* strains were precipitated with TCA and analysed by SDS-PAGE. As shown in Figure 6(a), compared with the B1 strain, the extracellular protein products were significantly more abundant in the Δ*flhF* mutant, although not as much as SCV1. The SCV1:*flhF* strain was found to be less abundant in secreted proteins, similar to the B1 or CΔ*flhF* strain. Furthermore, we quantified the extracellular protein levels by the BCA assay. As shown in Figure 6(b), the protein content in the supernatants of Δ*flhF* was approximately 2.2-fold higher than that of the B1 strain (*P <* 0.001), although slightly less than that of the SCV1. Importantly, extracellular secretion in SCV1 dropped back nearly to the levels of the B1 or CΔ*flhF* strain upon overexpression of *flhF*.

### *Downregulation of* flhF *is responsible for the secretion of two known extracellular virulence proteins*

**Figure 7.**
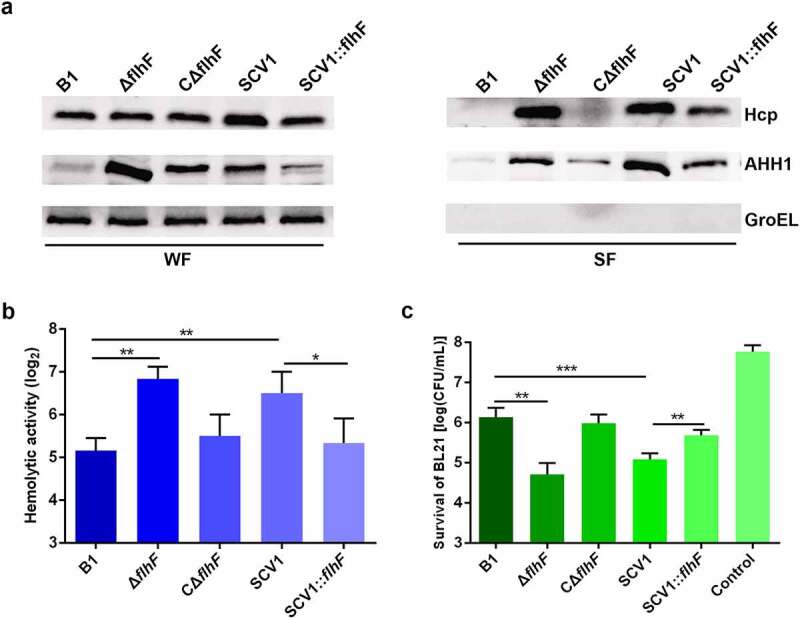
Downregulation of *flhF* is responsible for the secretion of haemolysin co-regulated protein (Hcp) and haemolysin (AHH1). (a) the expression and secretion of Hcp and AHH1 were determined by Western blot analysis. The samples were probed with anti-GroEL antibody as a cell lysis control for secreted fractions (SF) and a loading control for whole-cell fractions (WF). (b) the haemolytic activity of B1, Δ*flhF*, CΔ*flhF*, SCV1 and SCV1:*flhF*. The haemolytic activity is expressed as the fold dilution of the *A. hydrophila* culture filtrates that led to the lysis of 50% of the erythrocytes. (c) the antibacterial competition capability of B1, Δ*flhF*, CΔ*flhF*, SCV1 and SCV1:*flhF*. The antibacterial competition capability of the indicated strains against *E. coli* BL21 was defined as the amount of surviving *E. coli* after challenge. The values are the mean ± sd from three biological replicates. * *P* <0.05, ** *P* <0.01 or ***P <0.001.

To determine whether the lack of *flhF* altered the export of some known secretory factors, such as haemolysin coregulated protein (Hcp) and haemolysin (AHH1), the protein levels were assessed by Western blot analysis. The data showed that the levels of extracellular Hcp and AHH1 were higher in Δ*flhF* than in the B1 and CΔ*flhF* strains (Figure 7(a)). This result was consistent with the increase in total protein secretion observed for Δ*flhF*. Moreover, a significant reduction in the amount of the two proteins was observed in SCV1:*flhF* compared with SCV1, indicating that the downregulation of *flhF* is responsible for the enhanced protein secretion in SCV1.

As expected, the culture filtrates collected from Δ*flhF* exhibited higher haemolytic activity than those from the B1 strain. The enhanced haemolytic activity of SCV1 was almost completely restored with *flhF* overexpression (Figure 7(b)). Additionally, our previous report indicated that the export of Hcp contributes to the antibacterial competition capability of *A. hydrophila* [28]. As shown in Figure 7(c), similar to SCV1, coculturing *E. coli* BL21 with Δ*flhF* led to a one-log reduction in CFUs compared with coculturing *E. coli* with the B1 strain (*P <* 0.01). The competitive abilities of both SCV1 and Δ*flhF* were finally restored by the expression of *flhF*.

### The key residue of FlhF activity is crucial for phage resistance

**Figure 8.**
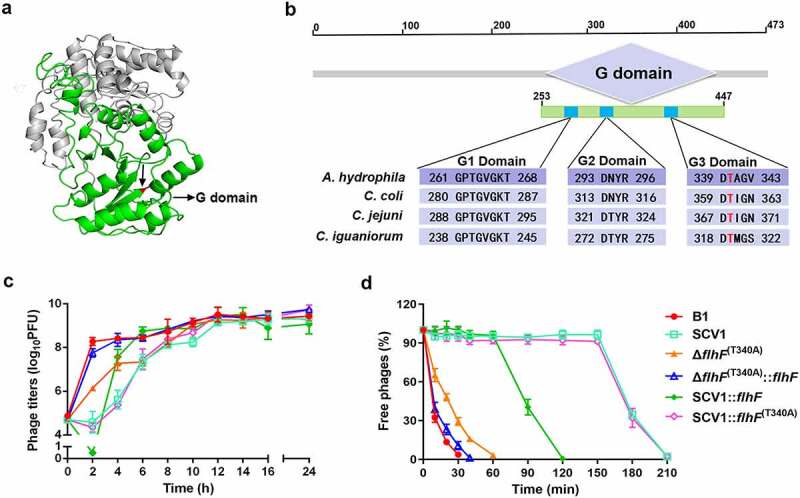
The key residue of FlhF activity is crucial for phage resistance. (a) the 3D structure of FlhF of *A. hydrophila* NJ-35. The G domain is shown in green and the Thr-340 residue is shown in red. (b) Clustal omega alignment of the G1, G2 and G3 subdomains of the FlhF protein orthologs from *A. hydrophila* NJ-35 (GenBank accession NZ_CP006870.1), *Campylobacter coli* RM4661 (GenBank accession CP007181.1), *C. jejuni* PT14 (GenBank accession NC_018709.4) and *Campylobacter iguaniorum* 1485E (GenBank accession CP009043.1). The G domain functions as a GTPase and contains multiple conserved subdomains, as indicated by G1, G2 and G3. (c) the titre of phage G65 cocultured with B1, SCV1, Δ*flhF* ^(t340a)^, Δ*flhF* ^(T340A)^:*flhF*, SCV1:*flhF* and SCV1:*flhF* ^(t340a)^. (d) Adsorption kinetics of G65 to the indicated strains. Δ*flhF* ^(t340a)^: a strain with a mutation in the Thr-340 residue of FlhF; Δ*flhF* ^(T340A)^::*flhF*: a strain with the overexpression of *flhF* in Δ*flhF* ^(t340a)^; SCV1:*flhF* ^(t340a)^: a strain with the overexpression of *flhF* ^(t340a)^ in SCV1.

Next, we aimed to investigate the molecular basis of *flhF*-involved phage resistance in the SCV1 strain. FlhF contains a basic *N*-terminal domain followed by a GTP-binging domain (G domain) (Figure 8(a)), which is required for the GTPase activity of FlhF [29]. By BLAST alignments, the G domain of FlhF in *A. hydrophila* was found to share conserved amino acid residues with *Campylobacter* spp. (Figure 8(b)). Thr-340 has been reported to be necessary for the activity of FlhF in *C. jejuni* [25]. To determine whether *flhF*-involved phage resistance in SCV1 was related to FlhF activity, we constructed a mutation Δ*flhF* ^(T340A)^ that creates a T340A substitution in the G3 domain of FlhF (Figure S3). As shown in Figure 8(c), the titre of phages propagated in Δ*flhF* ^(T340A)^ was consistent with that in Δ*flhF* but obviously lower than that in the B1 strain. Interestingly, the phage titre was restored to the same level as that in the B1 strain when complemented with *flhF* in Δ*flhF* ^(T340A)^. The data indicated that Thr-340, which is the key residue for FlhF activity, was necessary for phage resistance. Furthermore, we also found that although there was no significant difference in phage titre between the SCV1:*flhF* ^(T340A)^ and SCV1 strains (Figure 8(c)), complementing SCV1 with *flhF* but not *flhF* ^(T340A)^ significantly restored the adsorption rate (Figure 8(d)). This finding indicated that enhanced phage resistance of the SCV1 strain was closely associated with the activity of FlhF. To explore whether *A. hydrophila* underwent genetic modifications of *flhF* within *T. thermophila*, we sequenced the gene in both the B1 and SCV1 strains. *T. thermophila* predation did not cause any mutations in the *flhF* gene. However, notably, *flhF* remained at a constantly lower mRNA level in SCV1 than in B1 during phage infection (Figure S4).

## Discussion

Bacteria in the environment must survive predation from bacteriophages, heterotrophic protists, and predatory bacteria. This selective pressure could result in the evolution of a variety of defence mechanisms [30]. Our previous study indicated that the predator–prey interaction between *T. thermophila* and *A. hydrophila* caused the bacteria to evolve and display remarkable phage resistance [12], but the resistance mechanism is not yet clear.

In the present study, we performed high-throughput whole genome sequencing and RNA-Seq, and explored the altered genes related to biological changes in response to *T. thermophila* predation. The critical first step in a bacteriophage lytic cycle is attachment to the specific receptor on the bacterial surface, so mutation of the receptor is the most common cause for phage-resistance of the host. However, among 38 site variations identified in the SCV1 strain (Figure 1(a), Table S3), none occurs in previously reported phage receptors, such as outer membrane proteins (OMPs), lipopolysaccharides (LPS), pilus or capsular proteins [31], indicating that the lowered phage adsorption induced by *T. thermophila* predation might not be due to the alteration of phage receptors. Also, we identified six mutated genes which were downregulated or upregulated in SCV1 (Figure 1(b)), but this transcriptional alteration was not associated with the phage resistance phenotype of SCV1, since complementation of any of the six genes did not improve the sensitivity of SCV1 to phages (Figure 1(c)). Also, we complemented the other 24 mutated genes without alteration on mRNA level into the SCV1 strain, and similarly, phage sensitivity has been restored (Figure S2). These findings indicated that mutations in the genome do not contribute to the phage resistance phenotype in SCV1.

Further, our transcriptome analysis revealed 695 differentially expressed genes related to metabolism, biological regulation, locomotion, and transporter activity (Figure 2(a), Table S4). The mass sequence data suggest that the mechanisms of *A. hydrophila* evolution in response to *Tetrahymena* predation is multi-functional and involves multiple pathways. Notably, flagellar assembly, one of the top three enriched pathways, was significantly downregulated in SCV1. A total of 22 genes were identified to be associated with flagellar biosynthesis. Among them, the downregulation of *flhF*, a gene encoding a signal recognition particle (SRP)-type GTPase, has attracted our attention. *flhF* has been recognized as a global regulator that is linked to a range of flagellar gene expression, assembly, and function in various bacteria, such as *Pseudomonas* [32], *Vibrio* [33], *Campylobacter* [34], and *Shewanella* [35]. Also, the inactivation of *flhF* has been reported to cause a reduction in the phage adsorption of *C. jejuni* [25]. Consistent with these findings, our results demonstrated that the deletion of *flhF* has a consequence on non-motile and flagella-free phenotypes and enhanced phage resistance in *A. hydrophila*. Studies have demonstrated that phages could use the flagella as cell surface receptors for infection, although different manners for bacteria-phage interaction via flagella have been proposed. In *Caulobacter crescentus*, phages Cb13 and CbK have been shown to actively interact with the flagellum via the phage head filament and subsequently attach to receptors on the bacterial cell surface [36]. In *S*.Typhimurium, the phage iEPS5 injects its DNA through the flagellar filaments into the interior of the bacteria only when the bacterial flagella rotate [27]. In particular, a study on *C. jejuni* has revealed that the lowered phage adsorption exhibited by the *flhF* mutant was due to the absence of flagella [25]. Therefore, we speculated that phage resistance of the SCV1 strain may be related to impaired flagella. Unexpectedly, our study showed that *flhF* was involved in lowered phage susceptibility induced by *T. thermophila* predation (Figure 3(d)); however, this effect was independent of the function of FlhF, which positively regulates flagellar synthesis, as the overexpression of *flhF* in SCV1 did not apparently restore the flagellum and motility (Figure 4(a,c)). Moreover, the deletion of flagellin subunits FlaA and FlaB also revealed no detectable effect on the phage titres of the B1 strain (Figure 4(d,e)), indicating that the flagellar structure does not seem to be associated with the kinetics of phage infection.

Notably, the significantly altered expression of flagellins FlaA and FlaB described for the Δ*flhF* appears not to be applicable to the SCV1. FlaA in SCV1 was truncated, while FlaB was largely unaffected; neither FlaA nor FlaB was detected in the culture supernatants of SCV1 (Figure 4(b)). Therefore, it is reasonable to presume that the absence of flagellar motility in the SCVs may be due to truncated expression of FlaA or complete loss of FlaA and FlaB secretion. The truncated expression observed in FlaA may be interpreted with nonglycosylated flagellin. Similar phenomenon has been reported in *Helicobacter pylori*, in which destruction of glycosylation led to a decrease in the molecular weight of flagellin and resulted in the defect in flagellar biosynthesis [37]. This idea was further supported by the evidence that one site of the *pseI* gene, which has been shown to be required for flagellin glycosylation in *A. hydrophila* [38], was substituted in SCV1 based on genome sequencing analysis. A cytosine to thymine substitution at nucleotide 211 resulted in premature termination of PseI translation (Figure S5). This finding may theoretically account for the truncated expression observed in FlaA. Nevertheless, the functional significance of this single point mutation in PseI requires further experimental study.

Based on the adsorption kinetics assays from this study and our previous study [12], adsorption inhibition is a potential cause of phage resistance. Phage adsorption is a crucial step in the process of phage infection, as phage particles must find the suitable receptor among thousands of cell surface components [39]. In this study, TEM observation revealed that SCV1 was covered by a dense coating of surface-associated components (Figure 4(c)). It has been reported that the production of extracellular polymers can promote bacterial survival in harsh environments and, in some situations, provide a physical barrier between phages and their receptors [18]. We speculate that excessive extracellular proteins wrapped around the surface of SCV1 might prevent the phage from binding to its surface receptor by providing a physical barrier, as we found that SCV1 strain only showed the delayed phage adsorption rather than complete blockade. To demonstrate our assumption, we treated SCV1 cells with proteinase K or NaIO_4_ to degrade bacterial surface components and performed a phage sensitivity assay. Our data indicated that the reduced phage susceptibility of SCV1 was mostly related to the excessive surface proteins (Figure 5(c)). However, it should be noted that the reduction of phage titre was obviously observed in B1 strain after NaIO_4_ treatment, indicating that lipopolysaccharide may play an important role in the phage absorption. This seems to be inconsistent with the finding that NaIO_4_ treatment did not change the phage resistance of SCV1. The reason for this rather contradictory result may be due to the fact that the excessive extracellular proteins block the destructive effect of NaIO_4_ on the phage receptor of SCV1. Once the proteins coated around the surface of SCV1 are removed by protease K, the receptor might be exposed, which led to a significant increase in phage absorption.

In this study, we did not identify which proteins are involved in phage resistance of SCV1. We hypothesize that adsorption inhibition of phages was most likely due to the protective shield provided by various extracellular proteins, as we found that the secretory proteins with different molecular weights in the SCV1 strain were more abundant than those in the B1 strain, as revealed by analysis of the protein profile (Figure 6(a)) and BCA assay (Figure 6(b)). To further explore whether the blocking effect of extracellular proteins is specific or non-specific, we added a series of concentrations of bovine serum albumin (BSA) to the culture of B1 strain and evaluated the phage adsorption ability. Our data showed that BSA did not significantly affect the adsorption of phage to B1 strain; further TEM observation revealed no appearance of protein-coated bacteria (Figure S6). Therefore, current experimental data are not sufficient to allow a conclusion to be reached regarding the specific or non-specific block of extracellular proteins to phage adsorption. A previous study on *S. aureus* has reported that some macromolecular proteins (molecular weight＞1.5 MDa) in bovine serum could adhere to the bacterial cell surface and thereby sterically block phage adsorption, but the exogenously added BSA could not [40]. Determining the protein components involved in the phage resistance of SCV strain will provide insight into the defence mechanisms by which bacteria have developed against phage infection, and may uncover new avenues to develop a phage-based therapeutic method. In future study, we will manage to identify the proteins that have been coated on the surface of bacteria and reveal their role in blocking the phage adsorption.

Previous studies showed that the *flhF* gene was involved in protein secretion in several bacteria, such as *Pseudomonas putida* [41] and *Bacillus cereus* [42]. Depletion of *flhF* in *Bacillus subtilis* [43] and *E. coli* [44] led to an enhanced level of extracellular proteins. Here, the *flhF* mutant of *A. hydrophila* displayed increased levels of extracellular proteins, which further supports the involvement of FlhF in the regulation of protein secretion. As a flagellar biosynthesis regulator, *flhF* has been found to negatively regulate the protein secretion in *B. cereus*, such as haemolysin BL (HBL), phosphatidylcholine-preferring phospholipase C (PC-PLC), cytotoxin K, bacillolysin, nonhemolytic enterotoxin (NHE), and cereolysin O [42,45]. In accordance with this observation, our study demonstrated that deletion of *flhF* caused significantly enhanced secretion of haemolysin co-regulated protein (Hcp), a hallmark protein of type VI secretion system (T6SS) [46]. This finding indicates that FlhF might exert a regulatory function on the T6SS. Further investigations are required to characterize this association.

To date, the mechanism by how FlhF is involved in the regulation of protein secretion in *A. hydrophila* remains unknown. Nevertheless, it appears that enhanced secretion caused by the downregulation of *flhF* in SCV1 may be due to its role as an SRP-like protein, as the observed significant increase in protein secretion in SCV1 could be restored with FlhF, but not with FlhF with a T340A substitution in the G3 domain, which has been demonstrated to be a key residue for GTPase activity [25]. Also, an effect of FlhF downregulation on regulatory pathways shared between the genes encoding the altered proteins cannot be excluded. In agreement with what was observed in protein secretion, enhanced phage resistance of SCV1 was associated with the activity of FlhF, since no significant difference in phage titre was found between SCV1:*flhF* ^(T340A)^ and SCV1 strains, whereas complementing SCV1 with *flhF* but not with *flhF* ^(T340A)^ significantly restored the adsorption rate (Figure 8(c,d)). This finding further supports the idea that altered protein secretion due to downregulation of *flhF* is involved in phage resistance of SCV1. However, it must be pointed out that downregulation of *flhF* may not be entirely responsible for the enhancement of phage resistance in SCV1, as the increase in protein secretion and phage resistance observed in Δ*flhF* was not as much as that in SCV1, and overexpression of *flhF* in SCV1 only partially restored phage susceptibility. In this regard, it may be of interest to further evaluate the contribution of factors other than FlhF.

In summary, our results introduced *T. thermophila* predation as a powerful ecological agent in the survival of *A. hydrophila* and identified a phage resistance mechanism under the predatory pressure. Although this study was confined to examination of a single predator species, our findings may have implications for the function of ecosystems and the evolution of pathogens. Our results explain, at least in part, the phage defence mechanism of *A. hydrophila* in response to protistan predation, which has great significance for understanding how *A. hydrophila* persists and diversifies in complicated and changeable environments.

## Supplementary Material

Supplemental Material

## Data Availability

The genome sequence data and the RNA-Seq data generated in this study have been deposited in the National Center for Biotechnology Information (NCBI) Sequence Read Archive (SRA) under BioProject accession number PRJNA764348 and PRJNA764318.
