# Supplementary material for: Comparative transcriptome combined with morphophysiological analyses revealed the molecular mechanism underlying Tetrahymena thermophila predation-induced antiphage defense in Aeromonas hydrophila
Source: Virulence. 2022 Sep 24;13(1):1650–65. doi: 10.1080/21505594.2022.2127186 (PMC9518995; doi:10.1080/21505594.2022.2127186)
Supplement: Supplemental Material [file KVIR_A_2127186_SM5400.zip › supplementary/Figure S2.doc]

**Figure S2** The titer of phage G65 cocultured with B1, SCV1 and the strains that complementation of the corresponding genes in SCV1. The values are the mean ± SD from three biological replicates. ** *P* < 0.01.
