# Supplementary material for: Comparative transcriptome combined with morphophysiological analyses revealed the molecular mechanism underlying Tetrahymena thermophila predation-induced antiphage defense in Aeromonas hydrophila
Source: Virulence. 2022 Sep 24;13(1):1650–65. doi: 10.1080/21505594.2022.2127186 (PMC9518995; doi:10.1080/21505594.2022.2127186)
Supplement: Supplemental Material [file KVIR_A_2127186_SM5400.zip › supplementary/Figure S3.doc]

**Figure S3** **The nucleotide sequence comparison of *flhF* between B1 and SCV1 strains.** The sequence similarity was analyzed by BLAST program (https://blast.ncbi.nlm.nih.gov/Blast.cgi). The key active site of *flhF* was shown in red font.
