# Supplementary material for: Comparative transcriptome combined with morphophysiological analyses revealed the molecular mechanism underlying Tetrahymena thermophila predation-induced antiphage defense in Aeromonas hydrophila
Source: Virulence. 2022 Sep 24;13(1):1650–65. doi: 10.1080/21505594.2022.2127186 (PMC9518995; doi:10.1080/21505594.2022.2127186)
Supplement: Supplemental Material [file KVIR_A_2127186_SM5400.zip › supplementary/Figure S4.doc]

**Figure S4 The** **relative mRNA expression level of *flhF* in the B1 and SCV1 during phage infection.** Logarithmic-phase bacterial cultures were diluted to 5×10^6^ CFU/mL. Phage G65 was added to give a final concentration of 5×10^4^ PFU/mL and the cocultures were incubated at 28°C for 4 h with 180 rpm shaking. RNA was extracted every one hour interval. Bacteria cultured alone (without phage) for 4 h served as a control.
