# Supplementary material for: Comparative transcriptome combined with morphophysiological analyses revealed the molecular mechanism underlying Tetrahymena thermophila predation-induced antiphage defense in Aeromonas hydrophila
Source: Virulence. 2022 Sep 24;13(1):1650–65. doi: 10.1080/21505594.2022.2127186 (PMC9518995; doi:10.1080/21505594.2022.2127186)
Supplement: Supplemental Material [file KVIR_A_2127186_SM5400.zip › supplementary/Figure S6.doc]

**Figure S6 The effect of BSA on the susceptibility of B1 to phage G65.** (a) The titer of phage G65 after incubation with the B1 strain with 5mg/mL, 10mg/mL and 20mg/ mL BAS. (b) Adsorption kinetics of G65 to the B1 strain with 5mg/mL, 10mg/mL and 20mg/mL BAS. (c) TEM images of the B1 strain incubated in LB containing 5mg/mL, 10mg/mL and 20mg/mL BAS.
