# Supplementary material for: Comparative transcriptome combined with morphophysiological analyses revealed the molecular mechanism underlying Tetrahymena thermophila predation-induced antiphage defense in Aeromonas hydrophila
Source: Virulence. 2022 Sep 24;13(1):1650–65. doi: 10.1080/21505594.2022.2127186 (PMC9518995; doi:10.1080/21505594.2022.2127186)
Supplement: Supplemental Material [file KVIR_A_2127186_SM5400.zip › supplementary/Table S1.doc]

**Table S1** Strains and plasmids used in this study

| Strains or plasmids | Descriptions ^a^ | Sources |
| --- | --- | --- |
| Strains | | |
| A. hydrophila B1 | Culture alone of A. hydrophila NJ-35 non-exposed to T. thermophila | Collected in our laboratory |
| A. hydrophila SCV1 | Evolved strain of A. hydrophila NJ-35 after predation by T. thermophila | Collected in our laboratory |
| E. coli SM10 | E. coli strain, λpir^+^, Kan^r^ | Collected in our laboratory |
| *E.coli* BL21（DE3） | F^-^, ompT, hsdS (rB^-^mB^-^) gal, dcm（DE3） | TIANGEN |
| ΔflhF | B1 mutant with in-frame deletion of flhF, Amp^r^ | This study |
| CΔflhF | flhF gene complement strain; ΔflhF containing flhF-pMMB207, Amp^r^, Cm^r^ | This study |
| flhF^(T340A)^ | A T340A substitution in the G3 domain of the FlhF from B1 | This study |
| flhF^(T340A)^::flhF | flhF(T340A) mutant containing *f*lhF-pMMB207, Amp^r^, Cm^r^ | This study |
| SCV1::flhF | SCV1 containing flhF-pMMB207, Amp^r^, Cm^r^ | This study |
| Plasmids | | |
| pYAK1 | R6K-ori suicide vector, Cm^r^ | Collected in our laboratory |
| pET28a（+） | F1 origin, His tag, Kan^r^ | Novagen |
| flhF-pYAK1 | Plasmid pYAK1 carrying the two arms of flhF, Cm^r^ | This study |
| flhF^(T340A)^-pYAK1 | Plasmid pYAK1 carrying the flhF gene with a T340A substitution in the G3 domain and also the two arms of flhF, Cm^r^ | This study |
| pMMB207 | A shuttle plasmid with low-copy-number, Cm^r^ | Collected in our laboratory |
| flhF-pMMB207 | Plasmid pMMB207 carrying the complete ORF of *flhF*, Cm^r^ | This study |
| flhF-28a | Plasmid pET(+)-28a carrying the complete ORF of flhF, Cm^r^ | This study |
| aerA-28a | Plasmid pET(+)-28a carrying the complete ORF of aerA, Cm^r^ | This study |
| AHH1-28a | Plasmid pET(+)-28a carrying the complete ORF of AHH1, Cm^r^ | This study |

^a^ Cm^r^, chloramphenicol resistant; Amp^r^ Ampicillin; Kan^r^, kanamycin resistant.
