# Supplementary material for: Comparative transcriptome combined with morphophysiological analyses revealed the molecular mechanism underlying Tetrahymena thermophila predation-induced antiphage defense in Aeromonas hydrophila
Source: Virulence. 2022 Sep 24;13(1):1650–65. doi: 10.1080/21505594.2022.2127186 (PMC9518995; doi:10.1080/21505594.2022.2127186)
Supplement: Supplemental Material [file KVIR_A_2127186_SM5400.zip › supplementary/Table S2.doc]

**Table S2** Primers used in this study

| **Primer** | **Sequence (5’→3’)** | **Function** |
| --- | --- | --- |
| *lipA*-qPCR-F | CGTCCTCGGTGGGATAGTTGT | *lipA* expression analysis by qRT-PCR |
| *lipA*-qPCR-R | GGGCGGTCGCAGTTTCTT |  |
| *kdpD*-qPCR-F | CCTCCCGTCAGCGTCAACA | *kdpD* expression analysis by qRT-PCR |
| *kdpD*-qPCR-R | TCCCTGTCTGAGCACCTCTTC |  |
| *tctA*-qPCR-F | TCCTCCTGCCTGCGTTTGC | *tctA* expression analysis by qRT-PCR |
| *tctA*-qPCR-R | GCTCCTCGGTGATGGGTTTC |  |
| *eprA1*-qPCR-F | TGTGGGTGGACGGAGTGAGT | *eprA1* expression analysis by qRT-PCR |
| *eprA1*-qPCR-R | GGATGTCGCTCTTCTGGGTGT |  |
| *exbD*-qPCR-F | CAAGCCGCCCTTCATCGT | *exbD* expression analysis by qRT-PCR |
| *exbD*-qPCR-R | TGCATCGCCTCGGGAGTA |  |
| *cheW*-qPCR-F | TGCTGCTGGGTCAGTTCG | *cheW* expression analysis by qRT-PCR |
| *cheW*-qPCR-R | CACGGCAATCAGGACATCG |  |
| *flhF*-qPCR-F | CAGTTGGCCGAGGTGTTGA | *flhF* expression analysis by qRT-PCR |
| *flhF*-qPCR-R | TGGCGATGGTGGTGGTCT |  |
| *flgM*-qPCR-F | CAGCCAGGGTGCCAATAC | *flgM* expression analysis by qRT-PCR |
| *flgM*-qPCR-R | TGGTCAAGGAGACGGAGTC |  |
| *flaA*-qPCR-F | CATCACGGATACGGGAACG | *flaA* expression analysis by qRT-PCR |
| *flaA*-qPCR-R | GGACTGACTGGTGGCATTAGC |  |
| *flaB*-qPCR-F | CGGCAATTCCCGAAATACTGA | *flaB* expression analysis by qRT-PCR |
| *flaB*-qPCR-R | CCAAGCTGCTCGATGGCTCT |  |
| *fimC*-qPCR-F | ACAACCCGACTCCTTACTACA | *fimC* expression analysis by qRT-PCR |
| *fimC*-qPCR-R | GGTGACCGTCTGCTCTGA |  |
| *mutS*-qPCR-F | TGATGATGCGAATGACCTTGC | *mutS* expression analysis by qRT-PCR |
| *mutS*-qPCR-R | GCTCGACATCTCCCTGACCAA |  |
| *recA*-F | CGACCCCATCTATGCCGC | *recA* expression analysis by qRT-PCR |
| *recA*-R | CCATCTCACCTTCGATTTCCG |  |
| *flhF*-P1 | CAGGTCGACTCTAGAGGATCCCTCGCAGATTGGTGTCA | Construction of the *flhF* deletion mutant |
| *flhF*-P2 | GAGATGACGGCATGTATATGGATCAGGC |  |
| *flhF*-P3 | CATATACATGCCGTCATCTCCGCTAACT |  |
| *flhF*-P4 | GAGCTCGGTACCCGGGGATCCAGACCCTTGGCTACACAGT |  |
| *flhF*-a | TTCGCCCAGATTCAGACT | Identification of the *flhF* deletion mutant |
| *flhF*-b | ATGTCCAACAAGAAGGTCAC |  |
| *flhF*-CF | GAGCTCGGTACCCGGGGATCCGTGAAGATTAAGCGGTTTT | Construction of *flhF*-pMMB207 |
| *flhF*-CR | CAGGTCGACTCTAGAGGATCCTTACTCATAAAACTCCGAATC |  |
| AHH1-28a-P1 | ATGGGTCGCGGATCCGAATTCATGGATATTGGCGAACGTAC | Construction of AHH1 protein expression vector |
| AHH1-28a-P2 | TTGTCGACGGAGCTCGAATTCTCAGTGACTGGCCGGTG |  |
| *groEL*-28a-P1 | ATGGGTCGCGGATCCGAATTCATGGCAGCTAAAGAAGTCA | Construction of GroEL protein expression vector |
| *groEL*-28a-P2 | TTGTCGACGGAGCTCGAATTCTTACATCATGCCCATGC |  |
| *flaAB*-P1 | CAGGTCGACTCTAGAGGATCCACCAAGGCTATTCAGTCCC | Construction of the *flaAB* deletion mutant |
| *flaAB*-P2 | GAGAATAGTCGTGAGATCAGAATATGATGGTATAA |  |
| *flaAB*-P3 | CTGATCTCACGACTATTCTCCTAAGCTATTAAATTT |  |
| *flaAB*-P4 | GAGCTCGGTACCCGGGGATCC TCCATCCTGAGACGACCT |  |
| *flaAB*-a | TTGGCAGTTTCTGTCGC | Identification of the *flaAB* deletion mutant |
| *flaAB*-b | CGGTGGGTTGTCGTGTA |  |
| U876_15465-1 | GAGCTCGGTACCCGGGGATCCTTGGATGCCGTCACCAT | Construction of SCV1+p15465 complementary strain |
| U876_15465-2 | CAGGTCGACTCTAGAGGATCCTCAGACCGGATCTTCTTCC |  |
| U876_21325-1 | GAGCTCGGTACCCGGGGATCCATGAACAGTCTGCGACGG | Construction of SCV1+p21325 complementary strain |
| U876_21325-2 | CAGGTCGACTCTAGAGGATCCTCAATCCAGACTCTTCTTGAAG |  |
| U876_23735-1 | CAGGTCGACTCTAGAGGATCCTTACTTCTCGGTCAAGATCGG | Construction of SCV1+p21325 complementary strain |
| U876_23735-2 | GAGCTCGGTACCCGGGGATCCATGACCTATGACCCACTCGCT |  |
| U876_18065-1 | CAGGTCGACTCTAGAGGATCCTTACGGTTTCTGTTTGGTTTG | Construction of SCV1+p18065 complementary strain |
| U876_18065-2 | GAGCTCGGTACCCGGGGATCCATGCCCTTCCCTGAGAGG |  |
| U876_01305-1 | GAGCTCGGTACCCGGGGATCCTGAAAGACTTACTCAAGTTTTTGA | Construction of SCV1+p01305 complementary strain |
| U876_01305-2 | CAGGTCGACTCTAGAGGATCCTTACTCTTCGTCAAAACTGCC |  |
| U876_12715-1 | GAGCTCGGTACCCGGGGATCCATGAGCCAGCCCATGAGT | Construction of SCV1+p12715 complementary strain |
| U876_12715-2 | CAGGTCGACTCTAGAGGATCCTCAGTGGTTGAGATAGACATCCT |  |
| U876_19656-1 | CAGGTCGACTCTAGAGGATCCCTAACGCTTTGGCAAATGG | Construction of SCV1+p19656 complementary strain |
| U876_19656-2 | GAGCTCGGTACCCGGGGATCCATGCGTTCGCGTGTTTG |  |
| U876_19620-1 | CAGGTCGACTCTAGAGGATCCTCAGAGGCGCTTGGC | Construction of SCV1+p19620 complementary strain |
| U876_19620-2 | GAGCTCGGTACCCGGGGATCCATGGTTGCGGTTCGC |  |
| U876_21855-1 | CAGGTCGACTCTAGAGGATCCTTATTCGTAGTGGAACAGATCG | Construction of SCV1+p21855 complementary strain |
| U876_21855-2 | GAGCTCGGTACCCGGGGATCCATGTTCAAAAAGCTCAGAGGC |  |
| U876_23645-1 | CAGGTCGACTCTAGAGGATCCTTAAGTGAAATATTGCAAGAATC | Construction of SCV1+p23645 complementary strain |
| U876_23645-2 | GAGCTCGGTACCCGGGGATCCGTGACGAAAATCAAAAGTTTT |  |
| U876_06390-1 | CAGGTCGACTCTAGAGGATCCCTAGCCCCCCCGCTGA | Construction of SCV1+p06390 complementary strain |
| U876_06390-2 | GAGCTCGGTACCCGGGGATCCATGCAGCAGCCATTGGAA |  |
| U876_07085-1 | GAGCTCGGTACCCGGGGATCCATGACGACTTCTTCTGTTTCG | Construction of SCV1+p07085 complementary strain |
| U876_07085-2 | CAGGTCGACTCTAGAGGATCCTCAGGCTTTCGTCTTGTCG |  |
| U876_07930-1 | GAGCTCGGTACCCGGGGATCCATGAAACGCAGTGTGAAGCTG | Construction of SCV1+p07930 complementary strain |
| U876_07930-2 | CAGGTCGACTCTAGAGGATCCTTACTGCTGGCTTGCGTAGTA |  |
| U876_14230-1 | GAGCTCGGTACCCGGGGATCCATGCCTCAGTTCACTATTAATG | Construction of SCV1+p14230 complementary strain |
| U876_14230-2 | CAGGTCGACTCTAGAGGATCCTCATAAAAAAATATCTTCAAAACTG |  |
| U876_18155-1 | GAGCTCGGTACCCGGGGATCCATGGGTAACAACAAAAAAAGC | Construction of SCV1+p18155 complementary strain |
| U876_18155-2 | CAGGTCGACTCTAGAGGATCCCTAGAAGGGTTCCGGGATAA |  |
| U876_01025-1 | CAGGTCGACTCTAGAGGATCCTTATCCCTCTATGATGCGCA | Construction of SCV1+p01025 complementary strain |
| U876_01025-2 | GAGCTCGGTACCCGGGGATCCATGCAGACCCGCTACACC |  |
| U876_02865-1 | CAGGTCGACTCTAGAGGATCCTTAACCTGCTCTTACGGGTT | Construction of SCV1+p02865 complementary strain |
| U876_02865-2 | GAGCTCGGTACCCGGGGATCCATGCCAACAGTAACCTTCTAC |  |
| U876_06585-1 | GAGCTCGGTACCCGGGGATCCATGGGAAAACAGGGGCT | Construction of SCV1+p06585 complementary strain |
| U876_06585-2 | CAGGTCGACTCTAGAGGATCCCTAACCAACCCGAGCCA |  |
| U876_06950-1 | CAGGTCGACTCTAGAGGATCCTCAGGCGCGGATCAGC | Construction of SCV1+p06950 complementary strain |
| U876_06950-2 | GAGCTCGGTACCCGGGGATCCTTGTGCCTTTCCCCCC |  |
| U876_08670-1 | CAGGTCGACTCTAGAGGATCCTTATCCCTGTTGCTCGGTCT | Construction of SCV1+p08670 complementary strain |
| U876_08670-2 | GAGCTCGGTACCCGGGGATCCATGGCACTCGATTATTTTGC |  |
| U876_11590-1 | GAGCTCGGTACCCGGGGATCCGTGGCGCGCGGGG | Construction of SCV1+p11590 complementary strain |
| U876_11590-2 | CAGGTCGACTCTAGAGGATCCTTACTCCCAATTCGCCTTAT |  |
| U876_13585-1 | CAGGTCGACTCTAGAGGATCCTTAACCAGGCAACAATTCCA | Construction of SCV1+p13585 complementary strain |
| U876_13585-2 | GAGCTCGGTACCCGGGGATCCTTGCAGATACAAACGATGACA |  |
| U876_16610-1 | GAGCTCGGTACCCGGGGATCCATGAGCTACCGCGTTTTTT | Construction of SCV1+p16610 complementary strain |
| U876_16610-2 | CAGGTCGACTCTAGAGGATCCTTAGAAGCTGGCCCCCA |  |
| U876_17795-1 | CAGGTCGACTCTAGAGGATCCTTAAAAAGATTGACTTGTTTTTT | Construction of SCV1+p17795 complementary strain |
| U876_17795-2 | GAGCTCGGTACCCGGGGATCCATGAGTCAGAACAAGCGATAC |  |
| U876_17905-1 | CAGGTCGACTCTAGAGGATCCTTATGCTACTCGCTTACTCACA | Construction of SCV1+p17905 complementary strain |
| U876_17905-2 | GAGCTCGGTACCCGGGGATCCATGGACATTTTAGAGAATTTACTG |  |
| U876_18030-1 | CAGGTCGACTCTAGAGGATCCTCATTCATTGACTCCAGAGG | Construction of SCV1+p18030 complementary strain |
| U876_18030-2 | GAGCTCGGTACCCGGGGATCCATGAGCATCATGAATTATTGG |  |
| U876_18675-1 | CAGGTCGACTCTAGAGGATCCTCATACAAATCGCTCCACACT | Construction of SCV1+p18675 complementary strain |
| U876_18675-2 | GAGCTCGGTACCCGGGGATCCATGGCCAGAAAGGATGCC |  |
| U876_21680-1 | GAGCTCGGTACCCGGGGATCCGTGATCGGTGCCATGGTG | Construction of SCV1+p21680 complementary strain |
| U876_21680-2 | CAGGTCGACTCTAGAGGATCCTCACCCCCCCCCCATT |  |
| U876_06745-1 | GAGCTCGGTACCCGGGGATCCATGCGCCTGCCCCCC | Construction of SCV1+p06745 complementary strain |
| U876_06745-2 | CAGGTCGACTCTAGAGGATCCCTACTCCCCGCAGCCG |  |
| U876_16405-1 | GAGCTCGGTACCCGGGGATCCATGCGCTACCACATCAATG | Construction of SCV1+p16405 complementary strain |
| U876_16405-2 | CAGGTCGACTCTAGAGGATCCTCACGGCTTCCAGGAGT |  |
