# Supplementary material for: Comparative transcriptome combined with morphophysiological analyses revealed the molecular mechanism underlying Tetrahymena thermophila predation-induced antiphage defense in Aeromonas hydrophila
Source: Virulence. 2022 Sep 24;13(1):1650–65. doi: 10.1080/21505594.2022.2127186 (PMC9518995; doi:10.1080/21505594.2022.2127186)
Supplement: Supplemental Material [file KVIR_A_2127186_SM5400.zip › supplementary/Table S3.doc]

**Table S3 Mutations identified in the SCV1 strain compared to the B1 strain by whole-genome sequencing**

| **Function** | **Gene ID** | **Position** | **Reference allele** | **Allele variation** | **Variation type** | **Product** |
| --- | --- | --- | --- | --- | --- | --- |
| **Transport** | | | | | | |
| 1 | U876_06745 | 1436690 | - | G | Insertion | major facilitator transporter |
| 2 | U876_15465 | 3373557 | A | C | SNP | *cvrA*, potassium/proton antiporter |
| 3 | U876_21325 | 4635581 | - | G | Insertion | ABC transporter |
| 4 | U876_23735 | 5127912 | - | C | Insertion | conjugal transfer protein |
| **Regulation** | | | | | | |
| 5 | U876_16405 | 3577796 | - | T | Insertion | transcriptional regulator |
| 6 | U876_18065 | 3937718 | - | T | Insertion | AraC family transcriptional regulator |
| **Cell division/Replication** | | | | | | |
| 7 | U876_01305 | 284535 | A | G | SNP | DNA-directed RNA polymerase subunit beta' |
| 8 | U876_12715 | 2750053 | - | C | Insertion | cell division inhibitor |
| 9 | U876_19565 | 4260446 | - | T | Insertion | peptidoglycan-binding protein |
| 10 | U876_19620 | 4271991 | C | T | SNP | GTP diphosphokinase |
| 11 | U876_21855 | 4749409 | C | G | SNP | rod shape-determining protein |
| 12 | U876_23645 | 5111481 | - | T | Insertion | transcriptional antiterminator |
| **Metabolism** | | | | | | |
| 13 | U876_06390 | 1347552 | G | - | Deletion | *aceK*, isocitrate dehydrogenase |
| 14 | U876_07085 | 1524315 | G | A | SNP | tyrosine protein kinase |
|  |  | 1525185 | G | A | SNP |  |
| 15 | U876_07930 | 1719138 | - | CG | Insertion | c-type cytochrome |
|  |  | 1719139 | - | C |  |  |
|  |  | 1719143 | - | CA |  |  |
|  |  | 1719145 | - | AC |  |  |
|  |  | 1719148 | - | TA |  |  |
|  |  | 1868416 | - | GG |  |  |
| 16 | U876_14230 | 3099998 | C | T | SNP | *pseI*, pseudaminic acid synthase |
| 17 | U876_18155 | 3955685 | - | A | Insertion | polyphosphate kinase |
| **Unspecified** | | | | | | |
| 18 | U876_01025 | 227843 | - | C | Insertion | hypothetical protein |
| 19 | U876_02865 | 288880 | A | - | Deletion | hypothetical protein |
| 20 | U876_06585 | 1393082 | - | GGCAG | Insertion | hypothetical protein |
| 21 | U876_06950 | 1481298 | - | G | Insertion | hypothetical protein |
| 22 | U876_08670 | 1894371 | - | A | Insertion | hypothetical protein |
| 23 | U876_11590 | 2508928 | C | T | SNP | hypothetical protein |
| 24 | U876_13585 | 2968570 | - | T | Insertion | hypothetical protein |
| 25 | U876_16610 | 3625122 | - | A | Insertion | hypothetical protein |
|  |  | 3625125 | T | G | SNP |  |
| 26 | U876_17795 | 3863197 | - | C | Insertion | hypothetical protein |
| 27 | U876_17905 | 3896987 | - | A | Insertion | hypothetical protein |
| 28 | U876_18030 | 3926276 | - | T | Insertion | hypothetical protein |
| 29 | U876_18675 | 4067371 | A | - | Deletion | hypothetical protein |
| 30 | U876_21680 | 4711572 | - | C | Insertion | hypothetical protein |
