# Supplementary material for: Comparative transcriptome combined with morphophysiological analyses revealed the molecular mechanism underlying Tetrahymena thermophila predation-induced antiphage defense in Aeromonas hydrophila
Source: Virulence. 2022 Sep 24;13(1):1650–65. doi: 10.1080/21505594.2022.2127186 (PMC9518995; doi:10.1080/21505594.2022.2127186)
Supplement: Supplemental Material [file KVIR_A_2127186_SM5400.zip › supplementary/Table S4.doc]

**Table S4 Differentially expressed genes between SCV1 and B1**

| **Gene ID** | **Fold Change** | **P value** | **Up/Down** | **Product** |
| --- | --- | --- | --- | --- |
| U876_00035 | 2.029 | 0.000191 | Up | heat-shock protein |
| U876_00040 | 2.101 | 0.034684 | Up | heat shock protein IbpA |
| U876_00050 | 2.869 | 0.002659 | Up | hypothetical protein |
| U876_00070 | 3.789 | 2.11E-26 | Up | histidine kinase |
| U876_00075 | 21.351 | 1.32E-25 | Up | ATPase |
| U876_00085 | 14.276 | 3.92E-77 | Up | potassium-transporting ATPase subunit B |
| U876_00090 | 14.008 | 7.83E-32 | Up | ATPase |
| U876_00225 | 2.231 | 0.007643 | Up | membrane protein |
| U876_00240 | 2.723 | 6.40E-08 | Up | membrane protein |
| U876_00410 | 2.657 | 1.47E-09 | Up | sulfur acceptor protein CsdL |
| U876_00545 | 2.338 | 4.20E-06 | Up | phosphonate ABC transporter permease |
| U876_00635 | 4.445 | 6.76E-57 | Up | 50S ribosomal protein L31 |
| U876_00645 | 2.170 | 1.49E-05 | Up | malate dehydrogenase |
| U876_00725 | 2.809 | 5.78E-45 | Up | glutamine synthetase |
| U876_00770 | 3.071 | 4.28E-53 | Up | hypothetical protein |
| U876_00805 | 2.329 | 1.77E-27 | Up | regulator of ribonuclease activity A |
| U876_00860 | 3.135 | 2.13E-09 | Up | ATP-dependent protease subunit HslV |
| U876_00985 | 2.035 | 9.28E-16 | Up | ArgR family transcriptional regulator |
| U876_01060 | 2.473 | 9.10E-22 | Up | sodium:dicarboxylate symporter |
| U876_01065 | 2.846 | 1.38E-23 | Up | CoA-disulfide reductase |
| U876_01085 | 2.309 | 1.01E-23 | Up | protein disaggregation chaperone |
| U876_01090 | 2.617 | 0.045301 | Up | metallo-beta-lactamase |
| U876_01170 | 2.076 | 6.22E-06 | Up | hypothetical protein |
| U876_01230 | 2.815 | 3.20E-07 | Up | potassium transporter KtrA |
| U876_01280 | 2.442 | 1.77E-32 | Up | 50S ribosomal protein L11 |
| U876_01305 | 2.367 | 2.53E-36 | Up | DNA-directed RNA polymerase subunit beta' transposase |
| U876_01310 | 3.998 | 6.70E-19 | Up | transposase |
| U876_01360 | 2.008 | 6.59E-05 | Up | D,D-heptose 1,7- bisphosphate phosphatase |
| U876_01430 | 3.366 | 0.006494 | Up | short-chain dehydrogenase |
| U876_01450 | 2.018 | 2.61E-09 | Up | amino acid ABC transporter substrate-binding protein |
| U876_01475 | 2.539 | 1.17E-30 | Up | AraC family transcriptional regulator |
| U876_01170 | 2.076 | 6.22E-06 | Up | hypothetical protein |
| U876_01230 | 2.815 | 3.20E-07 | Up | potassium transporter KtrA |
| U876_01280 | 2.442 | 1.77E-32 | Up | 50S ribosomal protein L11 |
| U876_01305 | 2.367 | 2.53E-36 | Up | DNA-directed RNA polymerase subunit beta' transposase |
| U876_01310 | 3.998 | 6.70E-19 | Up | transposase |
| U876_01360 | 2.008 | 6.59E-05 | Up | D,D-heptose 1,7- bisphosphate phosphatase |
| U876_01430 | 3.366 | 0.006494 | Up | short-chain dehydrogenase |
| U876_01450 | 2.018 | 2.61E-09 | Up | amino acid ABC transporter substrate-binding protein |
| U876_01475 | 2.539 | 1.17E-30 | Up | AraC family transcriptional regulator |
| U876_01540 | 4.459 | 9.36E-15 | Up | hypothetical protein |
| U876_01615 | 2.209 | 1.56E-14 | Up | diguanylate cyclase |
| U876_01630 | 2.287 | 1.39E-20 | Up | hypothetical protein |
| U876_01745 | 3.300 | 3.49E-10 | Up | hypothetical protein |
| U876_01970 | 2.352 | 1.44E-10 | Up | tetrapyrrole methylase |
| U876_02030 | 2.663 | 7.17E-39 | Up | cell division protein FtsQ |
| U876_02140 | 2.363 | 4.70E-22 | Up | diguanylate cyclase |
| U876_02220 | 2.421 | 0.002719 | Up | tRNA-Phe |
| U876_02230 | 2.645 | 0.000392 | Up | tRNA-Phe |
| U876_02260 | 2.630 | 5.66E-23 | Up | chemotaxis protein CheV |
| U876_02285 | 2.061 | 7.11E-20 | Up | bacterioferritin |
| U876_02355 | 3.868 | 7.60E-09 | Up | hypothetical protein |
| U876_02400 | 3.301 | 1.45E-18 | Up | hypothetical protein |
| U876_02440 | 2.357 | 7.72E-06 | Up | 16S rRNA methyltransferase |
| U876_02450 | 2.345 | 9.27E-32 | Up | diguanylate cyclase |
| U876_02460 | 2.262 | 1.83E-07 | Up | haloacid dehalogenase |
| U876_02465 | 2.971 | 8.23E-21 | Up | Fe-S cluster assembly protein HesB |
| U876_02540 | 2.519 | 4.08E-15 | Up | hypothetical protein |
| U876_02545 | 2.334 | 7.18E-09 | Up | hypothetical protein |
| U876_02585 | 5.889 | 7.16E-10 | Up | hypothetical protein |
| U876_02710 | 2.176 | 0.007862 | Up | molecular chaperone DnaJ |
| U876_02745 | 2.017 | 7.74E-11 | Up | phage-shock protein |
| U876_02750 | 2.209 | 0.000495 | Up | phage-shock protein |
| U876_02765 | 2.427 | 2.56E-07 | Up | ABC transporter substrate-binding protein |
| U876_03125 | 5.011 | 5.87E-36 | Up | DNA-binding protein |
| U876_03270 | 2.260 | 1.84E-12 | Up | chemotaxis protein |
| U876_03470 | 2.235 | 1.84E-21 | Up | hypothetical protein |
| U876_03510 | 2.502 | 3.25E-10 | Up | multicopper oxidase |
| U876_03570 | 2.782 | 0.004486 | Up | membrane protein |
| U876_03580 | 8.822 | 0.007916 | Up | FMN-dependent NADH-azoreductase |
| U876_03700 | 2.466 | 0.000637 | Up | hypothetical protein |
| U876_03710 | 2.059 | 2.08E-10 | Up | inorganic pyrophosphatase |
| U876_03875 | 2.131 | 7.09E-14 | Up | hypothetical protein |
| U876_03880 | 3.427 | 5.54E-28 | Up | iron-sulfur cluster insertion protein ErpA |
| U876_03950 | 2.880 | 2.80E-10 | Up | hypothetical protein |
| U876_03965 | 2.461 | 9.10E-23 | Up | UTP--glucose-1-phosphate uridylyltransferase |
| U876_03970 | 9.917 | 1.19E-47 | Up | competence protein ComEA |
| U876_04080 | 2.575 | 2.18E-32 | Up | hypothetical protein |
| U876_04185 | 2.225 | 4.13E-16 | Up | cold-shock protein |
| U876_04240 | 2.305 | 1.19E-23 | Up | glycoside hydrolase |
| U876_04615 | 2.810 | 5.76E-42 | Up | hypothetical protein |
| U876_04720 | 7.867 | 1.94E-91 | Up | diguanylate phosphodiesterase |
| U876_04845 | 2.247 | 0.005404 | Up | exodeoxyribonuclease VII small subunit |
| U876_04860 | 2.250 | 3.95E-11 | Up | hypothetical protein |
| U876_04870 | 2.878 | 3.16E-07 | Up | RNA-binding protein YhbY |
| U876_04940 | 2.019 | 7.69E-14 | Up | 30S ribosomal protein S15 |
| U876_04955 | 2.967 | 3.18E-41 | Up | diguanylate cyclase |
| U876_05040 | 2.088 | 0.002952 | Up | Pyoverdine biosynthesis protein |
| U876_05055 | 2.295 | 0.017547 | Up | glyoxalase |
| U876_05270 | 2.805 | 8.24E-37 | Up | nitroreductase |
| U876_05490 | 2.559 | 3.04E-18 | Up | shikimate kinase |
| U876_05585 | 4.205 | 0.003843 | Up | valyl-tRNA synthetase |
| U876_05645 | 3.628 | 4.71E-12 | Up | tRNA-Met |
| U876_05965 | 2.807 | 1.88E-16 | Up | beta-hexosaminidase |
| U876_06125 | 2.392 | 0.035375 | Up | hypothetical protein |
| U876_06170 | 2.293 | 2.10E-07 | Up | two-component hybrid sensor and regulator |
| U876_06360 | 2.076 | 3.52E-19 | Up | TorR family transcriptional regulator |
| U876_06525 | 2.537 | 3.92E-21 | Up | transcriptional regulator |
| U876_06530 | 2.256 | 4.92E-19 | Up | gamma-glutamylputrescine oxidoreductase |
| U876_06870 | 5.077 | 0.000156 | Up | glutathione peroxidase |
| U876_07040 | 2.670 | 0.000577 | Up | hypothetical protein |
| U876_07145 | 2.418 | 9.26E-08 | Up | cold-shock protein |
| U876_07150 | 2.231 | 0.001350 | Up | hypothetical protein |
| U876_07195 | 3.574 | 2.41E-36 | Up | fructose-6-phosphate aldolase |
| U876_07200 | 3.041 | 2.40E-28 | Up | hypothetical protein |
| U876_07210 | 2.139 | 1.35E-06 | Up | 16S rRNA pseudouridylate synthase |
| U876_07475 | 3.421 | 3.78E-09 | Up | diguanylate cyclase |
| U876_07485 | 2.248 | 0.006737 | Up | histidine kinase |
| U876_07605 | 2.776 | 1.12E-07 | Up | hypothetical protein |
| U876_07670 | 2.086 | 4.28E-05 | Up | transcriptional regulator |
| U876_07760 | 3.172 | 3.27E-06 | Up | integrase |
| U876_08020 | 2.297 | 0.03226 | Up | translation initiation factor SUI1 |
| U876_08175 | 2.330 | 5.42E-33 | Up | peptidase M35 |
| U876_08265 | 2.022 | 5.29E-23 | Up | structural toxin protein RtxA |
| U876_08270 | 2.005 | 1.44E-12 | Up | hypothetical protein |
| U876_08285 | 3.260 | 5.72E-40 | Up | hypothetical protein |
| U876_08465 | 16.191 | 6.15E-44 | Up | 4-hydroxyphenylpyruvate dioxygenase |
| U876_08470 | 8.956 | 1.09E-21 | Up | dioxygenase |
| U876_08475 | 5.771 | 7.97E-42 | Up | 2-keto-4-pentenoate hydratase |
| U876_08610 | 2.137 | 0.039525 | Up | electron transporter RsxA |
| U876_08650 | 2.281 | 1.09E-05 | Up | glutaredoxin |
| U876_08760 | 2.470 | 0.002520 | Up | hypothetical protein |
| U876_08995 | 3.562 | 0.044914 | Up | UV protection and mutation protein |
| U876_09165 | 2.393 | 3.46E-16 | Up | hypothetical protein |
| U876_09250 | 3.127 | 3.79E-11 | Up | purine permease |
| U876_09265 | 15.059 | 5.04E-13 | Up | hypothetical protein |
| U876_09270 | 17.543 | 3.0E-108 | Up | hypothetical protein |
| U876_09275 | 19.062 | 3.69E-44 | Up | hypothetical protein |
| U876_09280 | 2.870 | 7.91E-13 | Up | hypothetical protein |
| U876_09590 | 2.087 | 0.025683 | Up | chemotaxis protein CheW |
| U876_09605 | 2.561 | 0.013868 | Up | anti-anti-sigma factor family protein |
| U876_09610 | 2.637 | 8.95E-07 | Up | chemotaxis protein CheY |
| U876_10245 | 2.405 | 2.00E-15 | Up | hypothetical protein |
| U876_10515 | 2.553 | 3.14E-13 | Up | molybdopterin-binding oxidoreductase |
| U876_10525 | 3.540 | 5.23E-36 | Up | phosphodiesterase |
| U876_10530 | 3.710 | 1.61E-06 | Up | short-chain dehydrogenase |
| U876_10560 | 4.383 | 2.03E-06 | Up | lipid A biosynthesis acyltransferase |
| U876_10690 | 2.345 | 4.16E-13 | Up | dsDNA-mimic protein |
| U876_10755 | 4.660 | 7.89E-76 | Up | acetyl-CoA acetyltransferase |
| U876_10830 | 2.647 | 0.043788 | Up | hypothetical protein |
| U876_10865 | 5.122 | 7.64E-73 | Up | ornithine carbamoyltransferase |
| U876_10870 | 6.565 | 5.36E-13 | Up | diaminopropionate ammonia-lyase |
| U876_10875 | 4.517 | 9.92E-57 | Up | peptidase |
| U876_10880 | 3.443 | 1.41E-39 | Up | phenylhydantoinase |
| U876_10885 | 3.286 | 6.62E-30 | Up | carbamate kinase |
| U876_10895 | 2.817 | 1.04E-07 | Up | permease |
| U876_10900 | 2.442 | 1.19E-32 | Up | hypothetical protein |
| U876_10905 | 4.297 | 9.25E-47 | Up | hypothetical protein |
| U876_10920 | 2.384 | 4.89E-33 | Up | selenate reductase subunit YgfK |
| U876_10925 | 2.464 | 1.65E-11 | Up | chlorohydrolase |
| U876_10930 | 2.164 | 1.48E-17 | Up | oxidoreductase |
| U876_10940 | 2.428 | 3.02E-23 | Up | xanthine permease XanP |
| U876_10945 | 2.395 | 2.12E-25 | Up | guanine deaminase |
| U876_10955 | 2.292 | 2.55E-17 | Up | xanthine dehydrogenase |
| U876_10960 | 2.041 | 1.66E-07 | Up | xanthine dehydrogenase |
| U876_10965 | 3.264 | 3.08E-28 | Up | xanthine dehydrogenase |
| U876_11110 | 2.475 | 0.000855 | Up | hypothetical protein |
| U876_11210 | 2.841 | 1.30E-11 | Up | hypothetical protein |
| U876_11305 | 8.428 | 5.51E-48 | Up | hypothetical protein |
| U876_11345 | 3.043 | 0.016017 | Up | DNA-binding protein |
| U876_11410 | 2.249 | 0.010111 | Up | Rha family transcriptional regulator |
| U876_11430 | 16.903 | 1.84E-14 | Up | cytolytic delta-endotoxin (insecticidal protein) |
| U876_11435 | 2.651 | 4.46E-07 | Up | protein UmuC |
| U876_11440 | 2.659 | 0.000443 | Up | UV protection and mutation protein |
| U876_11455 | 2.317 | 4.42E-08 | Up | transcriptional regulator |
| U876_11555 | 2.509 | 0.004273 | Up | hypothetical protein |
| U876_11615 | 2.670 | 2.55E-34 | Up | hypothetical protein |
| U876_11770 | 3.024 | 5.45E-52 | Up | ribosomal protein L32p |
| U876_11800 | 2.533 | 1.39E-15 | Up | acyl carrier protein |
| U876_11895 | 2.542 | 1.58E-36 | Up | hypothetical protein |
| U876_11900 | 2.431 | 1.43E-17 | Up | TetR family transcriptional regulator |
| U876_11925 | 3.130 | 0.000113 | Up | hypothetical protein |
| U876_11930 | 4.524 | 0.007375 | Up | integrase |
| U876_12215 | 2.975 | 6.62E-23 | Up | ribonucleotide-diphosphate reductase subunit beta |
| U876_12220 | 3.376 | 5.66E-15 | Up | ferredoxin |
| U876_12335 | 2.079 | 3.89E-07 | Up | ATPase |
| U876_12340 | 2.278 | 1.02E-17 | Up | diguanylate cyclase |
| U876_12390 | 2.088 | 6.47E-05 | Up | permease |
| U876_12465 | 2.486 | 5.76E-23 | Up | peptidase M20 |
| U876_12530 | 2.187 | 1.82E-14 | Up | membrane protein |
| U876_12610 | 3.152 | 5.14E-06 | Up | antibiotic biosynthesis monooxygenase |
| U876_12640 | 2.493 | 2.18E-25 | Up | PHA granule-associated protein |
| U876_12655 | 2.707 | 7.61E-06 | Up | ribosome-associated protein |
| U876_12710 | 4.650 | 2.02E-15 | Up | hypothetical protein |
| U876_12810 | 275.648 | 9.3E-199 | Up | transporter |
| U876_12815 | 208.961 | 3.4E-134 | Up | TctB |
| U876_12820 | 90.523 | 5.68E-15 | Up | tripartite tricarboxylate transporter TctA |
| U876_12975 | 2.225 | 5.95E-06 | Up | AraC family transcriptional regulator |
| U876_12980 | 19.332 | 1.96E-14 | Up | 3-ketoacyl-ACP reductase |
| U876_13030 | 2.073 | 5.00E-26 | Up | pyruvate formate-lyase |
| U876_13080 | 2.348 | 0.001174 | Up | hypothetical protein |
| U876_13160 | 2.132 | 1.47E-05 | Up | 5'-nucleotidase |
| U876_13185 | 2.285 | 1.21E-07 | Up | 50S ribosomal protein L31 |
| U876_13300 | 55.704 | 3.74E-55 | Up | hypothetical protein |
| U876_13310 | 60.084 | 7.04E-12 | Up | pterin-4-alpha-carbinolamine dehydratase |
| U876_13315 | 47.053 | 1.58E-23 | Up | pterin-4-alpha-carbinolamine dehydratase |
| U876_13320 | 43.270 | 3.30E-12 | Up | phenylalanine 4-monooxygenase |
| U876_13405 | 2.052 | 6.04E-13 | Up | recombination factor protein RarA |
| U876_13450 | 3.622 | 8.82E-43 | Up | translation initiation factor IF-1 |
| U876_13465 | 2.282 | 1.80E-13 | Up | stationary phase/starvation inducible regulatory protein CspD |
| U876_13505 | 2.296 | 0.000478 | Up | hypothetical protein |
| U876_13510 | 2.605 | 1.86E-30 | Up | membrane protein |
| U876_13515 | 2.068 | 9.40E-25 | Up | type VI secretion protein IcmF |
| U876_13520 | 2.360 | 4.20E-30 | Up | type VI secretion protein |
| U876_13525 | 2.445 | 7.14E-27 | Up | type VI secretion protein |
| U876_13530 | 2.337 | 4.22E-30 | Up | ATPase AAA |
| U876_13610 | 3.114 | 1.51E-30 | Up | hypothetical protein |
| U876_13675 | 2.384 | 0.000835 | Up | membrane protein |
| U876_13780 | 2.029 | 0.007603 | Up | DNA-binding protein |
| U876_13900 | 3.996 | 1.83E-18 | Up | transposase |
| U876_13915 | 10.104 | 1.63E-15 | Up | ubiquitin--protein ligase |
| U876_14010 | 2.081 | 2.81E-09 | Up | tRNA (cytidine/uridine-2'-O-)-methyltransferase |
| U876_14015 | 2.567 | 1.77E-16 | Up | hypothetical protein |
| U876_14090 | 2.095 | 9.24E-09 | Up | hypothetical protein |
| U876_14100 | 2.174 | 0.002808 | Up | arsenate reductase |
| U876_14160 | 2.350 | 9.76E-30 | Up | hypothetical protein |
| U876_14165 | 4.132 | 6.14E-20 | Up | inositol monophosphatase |
| U876_14300 | 2.020 | 2.52E-08 | Up | hypothetical protein |
| U876_14490 | 2.793 | 6.64E-06 | Up | hypothetical protein |
| U876_14635 | 2.004 | 9.61E-15 | Up | membrane protein |
| U876_14895 | 2.164 | 1.65E-06 | Up | sorbose reductase |
| U876_14900 | 2.588 | 1.42E-12 | Up | ferredoxin |
| U876_14920 | 2.097 | 0.033939 | Up | ATP-dependent protease |
| U876_15010 | 2.260 | 6.29E-05 | Up | hypothetical protein |
| U876_15075 | 2.219 | 0.002116 | Up | peptidylprolyl isomerase |
| U876_15130 | 2.183 | 2.23E-06 | Up | membrane protein |
| U876_15170 | 2.013 | 1.03E-16 | Up | ferric uptake regulator |
| U876_15400 | 2.047 | 1.88E-07 | Up | chemotaxis protein |
| U876_15515 | 3.817 | 0.000186 | Up | pseudouridylate synthase |
| U876_15605 | 2.080 | 0.001677 | Up | membrane protein |
| U876_15670 | 7.406 | 2.37E-21 | Up | hypothetical protein |
| U876_15675 | 3.295 | 0.018707 | Up | hypothetical protein |
| U876_15855 | 2.231 | 0.048542 | Up | restriction endonuclease EcoEI subunit M |
| U876_16085 | 2.531 | 7.47E-26 | Up | membrane protein |
| U876_16285 | 2.293 | 7.70E-07 | Up | phosphodiesterase |
| U876_16355 | 3.043 | 3.88E-07 | Up | hemolysin D |
| U876_16360 | 2.099 | 9.18E-07 | Up | acriflavine resistance protein B |
| U876_16390 | 2.066 | 0.031657 | Up | iron ABC transporter substrate-binding protein |
| U876_16535 | 2.275 | 1.78E-06 | Up | N-acetylneuraminate lyase |
| U876_16620 | 4.545 | 6.49E-06 | Up | 23S rRNA methyltransferase |
| U876_16625 | 2.293 | 1.61E-13 | Up | peptidase P60 |
| U876_16670 | 3.693 | 2.14E-35 | Up | acetyltransferase |
| U876_16675 | 195.165 | 4.76E-141 | Up | peptidase M23 |
| U876_16710 | 2.170 | 0.036745 | Up | damage-inducible protein DinB |
| U876_16725 | 2.280 | 9.60E-12 | Up | hypothetical protein |
| U876_16730 | 2.528 | 2.14E-36 | Up | hypothetical protein |
| U876_16755 | 2.088 | 4.08E-05 | Up | transporter |
| U876_16760 | 2.323 | 9.14E-19 | Up | XRE family transcriptional regulator |
| U876_16765 | 2.443 | 7.76E-07 | Up | hypothetical protein |
| U876_16870 | 2.265 | 6.69E-10 | Up | peptidylprolyl isomerase |
| U876_16930 | 2.700 | 1.99E-28 | Up | aldehyde dehydrogenase |
| U876_17115 | 2.243 | 2.66E-15 | Up | potassium transporter |
| U876_17435 | 3.779 | 2.31E-32 | Up | hypothetical protein |
| U876_17440 | 3.021 | 2.14E-25 | Up | hypothetical protein |
| U876_17445 | 2.174 | 1.65E-20 | Up | hypothetical protein |
| U876_17515 | 2.141 | 7.38E-13 | Up | nucleotide-binding protein |
| U876_17565 | 2.800 | 6.89E-15 | Up | hypothetical protein |
| U876_17570 | 2.003 | 3.56E-05 | Up | transcriptional regulator |
| U876_17600 | 3.181 | 4.50E-44 | Up | hypothetical protein |
| U876_17605 | 2.835 | 8.93E-08 | Up | hypothetical protein |
| U876_17625 | 4.015 | 5.62E-10 | Up | cyclopropane-fatty-acyl-phospholipid synthase |
| U876_17630 | 3.013 | 0.001828 | Up | plasmid partition ParA protein |
| U876_17635 | 2.877 | 1.28E-06 | Up | FAD-dependent oxidoreductase |
| U876_17640 | 2.021 | 0.013741 | Up | short-chain dehydrogenase |
| U876_17645 | 2.923 | 0.000288 | Up | transcriptional regulator |
| U876_17650 | 2.716 | 5.09E-10 | Up | deoxyribodipyrimidine photolyase |
| U876_17655 | 4.733 | 1.40E-19 | Up | MerR family transcriptional regulator |
| U876_17660 | 3.511 | 3.42E-33 | Up | hypothetical protein |
| U876_17750 | 2.772 | 4.63E-30 | Up | hypothetical protein |
| U876_17860 | Inf | 0.037123 | Up | hypothetical protein |
| U876_17990 | 3.527 | 0.001550 | Up | hypothetical protein |
| U876_18155 | 2.556 | 1.55E-10 | Up | polyphosphate kinase |
| U876_18190 | 2.283 | 0.000577 | Up | hypothetical protein |
| U876_18250 | 8.705 | 3.20E-135 | Up | chemotaxis protein |
| U876_18255 | 3.099 | 1.45E-31 | Up | chemotaxis protein CheY |
| U876_18260 | 3.634 | 6.31E-22 | Up | anti-sigma B factor antagonist |
| U876_18265 | 4.580 | 1.38E-69 | Up | chemotaxis protein CheA |
| U876_18270 | 3.265 | 6.87E-47 | Up | chemotaxis protein |
| U876_18275 | 7.797 | 2.14E-46 | Up | chemotaxis protein CheW |
| U876_18280 | 3.578 | 3.64E-63 | Up | chemotaxis protein |
| U876_18285 | 3.995 | 3.27E-44 | Up | SAM-dependent methyltransferase |
| U876_18290 | 5.509 | 4.27E-43 | Up | chemotaxis protein CheD |
| U876_18295 | 4.416 | 3.18E-43 | Up | chemotaxis protein CheY |
| U876_18340 | 2.436 | 0.005544 | Up | ferredoxin |
| U876_18425 | 2.136 | 0.002906 | Up | hypothetical protein |
| U876_18440 | 3.397 | 8.45E-14 | Up | diguanylate cyclase |
| U876_18445 | 2.376 | 0.002805 | Up | hypothetical protein |
| U876_18515 | 2.437 | 0.028879 | Up | SAM-dependent methyltransferase |
| U876_18520 | 4.413 | 1.66E-69 | Up | chitinase |
| U876_18525 | 2.114 | 4.11E-21 | Up | peptidase M66 |
| U876_18530 | 2.382 | 2.09E-36 | Up | glycoside hydrolase |
| U876_18600 | 4.336 | 0.000474 | Up | membrane protein |
| U876_18710 | 4.339 | 0.004897 | Up | hypothetical protein |
| U876_18720 | 2.822 | 0.035479 | Up | hypothetical protein |
| U876_18725 | 3.147 | 0.031529 | Up | hypothetical protein |
| U876_18800 | 2.512 | 2.30E-12 | Up | hypothetical protein |
| U876_19050 | 2.025 | 2.33E-10 | Up | 50S ribosomal protein L21 |
| U876_19055 | 2.289 | 3.09E-16 | Up | octaprenyl diphosphate synthase |
| U876_19125 | 515.371 | 0 | Up | hypothetical protein |
| U876_19130 | Inf | 2.33E-30 | Up | DL-methionine transporter ATP-binding subunit |
| U876_19135 | Inf | 6.58E-38 | Up | methionine ABC transporter permease |
| U876_19140 | Inf | 1.26E-239 | Up | DL-methionine transporter substrate-binding subunit |
| U876_19145 | Inf | 1.03E-20 | Up | transcriptional regulator |
| U876_19150 | Inf | 7.59E-121 | Up | chemotaxis protein |
| U876_19155 | Inf | 4.11E-80 | Up | diguanylate phosphodiesterase |
| U876_19160 | Inf | 4.13E-134 | Up | histidine kinase |
| U876_19165 | Inf | 1.29E-71 | Up | hypothetical protein |
| U876_19385 | 2.119 | 6.53E-25 | Up | diguanylate phosphodiesterase |
| U876_19390 | 3.736 | 4.85E-49 | Up | molybdopterin-binding oxidoreductase |
| U876_19430 | 2.426 | 2.86E-11 | Up | multidrug resistance protein MdtH |
| U876_19495 | 2.217 | 1.91E-07 | Up | zinc-responsive transcriptional regulator |
| U876_19525 | 3.897 | 4.23E-18 | Up | transposase |
| U876_19545 | 2.307 | 5.40E-26 | Up | glutamyl-tRNA amidotransferase |
| U876_19550 | 3.563 | 2.05E-22 | Up | 30S ribosomal protein S21 |
| U876_19600 | 2.070 | 7.78E-05 | Up | cell division protein FtsB |
| U876_19740 | 2.944 | 2.62E-43 | Up | RNA polymerase sigma factor RpoE |
| U876_20160 | 4.112 | 3.35E-15 | Up | hypothetical protein |
| U876_20175 | 2.377 | 5.09E-24 | Up | 30S ribosomal protein S18 |
| U876_20180 | 2.467 | 1.77E-32 | Up | 30S ribosomal protein S6 |
| U876_20225 | 2.017 | 1.76E-16 | Up | diguanylate cyclase |
| U876_20250 | 2.614 | 4.78E-29 | Up | type IV pilin |
| U876_20255 | 2.026 | 1.31E-15 | Up | hypothetical protein |
| U876_20260 | 2.234 | 6.67E-16 | Up | type IV pilin |
| U876_20265 | 3.287 | 6.19E-29 | Up | type IV pilin |
| U876_20270 | 2.204 | 3.45E-05 | Up | type IV pilin |
| U876_20335 | 2.193 | 1.37E-18 | Up | 30S ribosomal protein S20 |
| U876_20345 | 2.917 | 7.42E-36 | Up | transcriptional regulator |
| U876_20545 | 2.180 | 0.001323 | Up | hypothetical protein |
| U876_20550 | 2.451 | 1.43E-06 | Up | hypothetical protein |
| U876_20580 | 3.092 | 3.50E-08 | Up | DEAD/DEAH box helicase |
| U876_20585 | 2.164 | 0.007260 | Up | ABC transporter substrate-binding protein |
| U876_20695 | 3.253 | 1.58E-30 | Up | N-acetylglucosamine-binding protein A |
| U876_20750 | 2.121 | 8.90E-10 | Up | hypothetical protein |
| U876_20955 | 2.855 | 0.028467 | Up | GCN5 family acetyltransferase |
| U876_21035 | 4.572 | 1.11E-39 | Up | hypothetical protein |
| U876_21050 | 2.682 | 0.000131 | Up | hypothetical protein |
| U876_21075 | 2.885 | 1.13E-06 | Up | DEAD/DEAH box helicase |
| U876_21095 | 2.915 | 0.001685 | Up | hypothetical protein |
| U876_21240 | 2.547 | 1.93E-13 | Up | hypothetical protein |
| U876_21290 | 3.487 | 2.04E-05 | Up | hypothetical protein |
| U876_21295 | 3.569 | 0.000788 | Up | hypothetical protein |
| U876_21355 | 2.493 | 9.61E-29 | Up | gamma-glutamyl-gamma-aminobutyrate hydrolase |
| U876_21435 | 2.275 | 0.000694 | Up | hypothetical protein |
| U876_21730 | 2.043 | 0.000153 | Up | acid phosphatase |
| U876_21745 | 5.147 | 4.77E-32 | Up | chemotaxis protein |
| U876_21750 | 5.038 | 1.44E-08 | Up | anti-anti-sigma regulatory factor |
| U876_21755 | 8.284 | 9.68E-23 | Up | histidine kinase |
| U876_21760 | 4.787 | 6.65E-40 | Up | chemotaxis protein CheY |
| U876_21765 | 4.867 | 7.77E-27 | Up | chemotaxis protein CheY |
| U876_21785 | 2.944 | 5.99E-15 | Up | hypothetical protein |
| U876_22230 | 2.180 | 1.31E-26 | Up | 30S ribosomal protein S13 |
| U876_22235 | 2.325 | 5.74E-35 | Up | preprotein translocase subunit SecY |
| U876_22370 | 2.914 | 1.74E-23 | Up | hypothetical protein |
| U876_22390 | 2.859 | 0.001849 | Up | competence protein ComF |
| U876_22405 | 2.046 | 1.23E-10 | Up | preprotein translocase subunit SecB |
| U876_22480 | 2.371 | 6.64E-06 | Up | D-tyrosyl-tRNA(Tyr) deacylase |
| U876_22550 | 2.316 | 4.99E-06 | Up | transcriptional regulator |
| U876_22565 | 2.185 | 9.08E-06 | Up | tRNA threonylcarbamoyl adenosine biosynthesis protein RimN |
| U876_22595 | 2.312 | 3.43E-11 | Up | peptide deformylase |
| U876_22600 | 2.449 | 3.89E-08 | Up | methionyl-tRNA formyltransferase |
| U876_22730 | 2.225 | 3.26E-19 | Up | polyketide cyclase |
| U876_22900 | 3.622 | 3.38E-39 | Up | cell division protein ZapB |
| U876_22945 | 2.228 | 5.82E-13 | Up | hypothetical protein |
| U876_23060 | 2.241 | 5.63E-06 | Up | 50S ribosomal protein L28 |
| U876_23115 | 3.000 | 7.20E-34 | Up | hypothetical protein |
| U876_23165 | 4.147 | 5.73E-74 | Up | acetoacetyl-CoA synthetase |
| U876_23195 | 2.362 | 2.44E-05 | Up | hypothetical protein |
| U876_23215 | 2.493 | 1.08E-16 | Up | isochorismatase |
| U876_23365 | 4.115 | 4.75E-09 | Up | hypothetical protein |
| U876_23390 | 2.037 | 1.12E-24 | Up | thioredoxin |
| U876_23435 | 2.134 | 4.83E-05 | Up | DNase TatD |
| U876_23480 | 4.312 | 1.53E-29 | Up | hypothetical protein |
| U876_24000 | 3.154 | 8.54E-07 | Up | DNA-binding protein |
| U876_24025 | 2.956 | 1.90E-10 | Up | conjugal transfer protein TraG |
| U876_24170 | 2.117 | 2.80E-08 | Up | transposase |
| U876_24440 | 2.330 | 0.012540 | Up | sulfite reductase |
| U876_24480 | 2.253 | 9.28E-06 | Up | ribonuclease P |
| U876_24485 | 3.095 | 1.55E-23 | Up | 50S ribosomal protein L34 |
| U876_00160 | 0.494 | 0.000585 | Down | phosphate starvation-inducible protein PsiE |
| U876_00340 | 0.173 | 0.048186 | Down | membrane protein |
| U876_00595 | 0.389 | 4.10E-08 | Down | acetolactate synthase catalytic subunit |
| U876_00600 | 0.475 | 0.002239 | Down | acetolactate synthase |
| U876_00670 | 0.206 | 5.93E-47 | Down | hypothetical protein |
| U876_00795 | 0.390 | 3.95E-06 | Down | hypothetical protein |
| U876_00825 | 0.165 | 0.010707 | Down | nitrite transporter NirC |
| U876_00870 | 0.484 | 4.01E-26 | Down | arginyl-tRNA synthetase |
| U876_01015 | 0.241 | 2.65E-62 | Down | chemotaxis protein |
| U876_01165 | 0.448 | 0.001012 | Down | sulfite oxidase subunit YedZ |
| U876_01600 | 0.078 | 6.50E-06 | Down | hypothetical protein |
| U876_01620 | 0.394 | 0.0033041 | Down | ligand-gated channel protein |
| U876_01655 | 0.436 | 0.019081 | Down | LuxR family transcriptional regulator |
| U876_01790 | 0.499 | 1.95E-11 | Down | ABC transporter permease |
| U876_01960 | 0.395 | 2.16E-20 | Down | hypothetical protein |
| U876_24505 | 0.480 | 6.99E-12 | Down | bacterial RNase P |
| U876_02280 | 0.473 | 0.013004 | Down | (2Fe-2S)-binding protein |
| U876_02300 | 0.231 | 2.91E-19 | Down | hypothetical protein |
| U876_02630 | 0.406 | 9.77E-09 | Down | dehydrogenase |
| U876_02685 | 0.499 | 0.017501 | Down | hypothetical protein |
| U876_02850 | 0.448 | 9.11E-19 | Down | DNA mismatch repair protein MutS |
| U876_02900 | 0.290 | 0.006929 | Down | tRNA-Ser |
| U876_03100 | 0.383 | 4.72E-20 | Down | alpha/beta hydrolase |
| U876_03225 | 0.459 | 1.50E-09 | Down | peptide ABC transporter substrate-binding protein |
| U876_03285 | 0.391 | 1.77E-40 | Down | cytochrome d terminal oxidase subunit 1 |
| U876_03290 | 0.391 | 2.87E-41 | Down | cytochrome d ubiquinol oxidase subunit 2 |
| U876_03295 | 0.347 | 1.51E-37 | Down | membrane protein |
| U876_03300 | 0.497 | 9.99E-16 | Down | hypothetical protein |
| U876_03315 | 0.485 | 3.92E-10 | Down | biopolymer transporter ExbD |
| U876_03465 | 0.197 | 9.74E-13 | Down | transporter |
| U876_03565 | 0.424 | 0.011434 | Down | glyoxalase |
| U876_03925 | 0.495 | 4.30E-12 | Down | membrane protein |
| U876_04105 | 0.455 | 0.027535 | Down | tRNA-Phe |
| U876_04215 | 0.449 | 3.62E-05 | Down | tRNA-Ala |
| U876_04220 | 0.472 | 1.25E-07 | Down | tRNA-Ala |
| U876_04225 | 0.326 | 0.005004 | Down | tRNA-Ala |
| U876_04230 | 0.309 | 0.001708 | Down | tRNA-Ala |
| U876_04285 | 0.470 | 0.000125 | Down | peptide ABC transporter permease |
| U876_04415 | 0.242 | 2.28E-85 | Down | cytochrome C biogenesis protein CcsA |
| U876_04420 | 0.293 | 3.99E-07 | Down | DNA/RNA endonuclease G |
| U876_04600 | 0.477 | 1.21E-14 | Down | chemotaxis protein |
| U876_04635 | 0.494 | 2.29E-16 | Down | signal peptidase |
| U876_04865 | 0.493 | 0.000289 | Down | hypothetical protein |
| U876_05235 | 0.450 | 2.94E-16 | Down | 2-octaprenyl-3-methyl-6-methoxy-1,4-benzoquinol hydroxylase |
| U876_05285 | 0.478 | 1.10E-13 | Down | glycerate dehydrogenase |
| U876_05290 | 0.333 | 0.004714 | Down | hypothetical protein |
| U876_05305 | 0.343 | 0.011376 | Down | hypothetical protein |
| U876_05470 | 0.392 | 2.94E-25 | Down | fimbrial protein |
| U876_06095 | 0.498 | 2.09E-05 | Down | tungsten ABC transporter substrate-binding protein |
| U876_06225 | 0.480 | 2.83E-11 | Down | cation:proton antiport protein |
| U876_06230 | 0.277 | 3.73E-46 | Down | gamma-glutamyltransferase |
| U876_06315 | 0.495 | 3.35E-13 | Down | 3-oxoacyl-ACP synthase |
| U876_06550 | 0.217 | 1.51E-06 | Down | phospho-2-dehydro-3-deoxyheptonate aldolase |
| U876_06615 | 0.161 | 1.96E-08 | Down | porin |
| U876_06620 | 0.357 | 3.39E-05 | Down | fructokinase |
| U876_06625 | 0.401 | 1.09E-05 | Down | PTS system sucrose-specific transporter subuits IIBC |
| U876_06655 | 0.254 | 0.033031 | Down | ArsR family transcriptional regulator |
| U876_06680 | 0.332 | 4.71E-38 | Down | diguanylate cyclase |
| U876_06690 | 0.392 | 1.19E-20 | Down | cytochrome D ubiquinol oxidase subunit II |
| U876_06765 | 0.414 | 0.013568 | Down | hypothetical protein |
| U876_06895 | 0.467 | 1.16E-11 | Down | indole-3-glycerol phosphate synthase |
| U876_06905 | 0.330 | 9.19E-09 | Down | anthranilate synthase subunit II |
| U876_06910 | 0.243 | 7.64E-20 | Down | anthranilate synthase component I |
| U876_07035 | 0.463 | 3.77E-20 | Down | UDP-phosphate alpha-N-acetylglucosaminyl 1-phosphate transferase |
| U876_07045 | 0.388 | 0.000793 | Down | Vi polysaccharide biosynthesis protein VipA/TviB |
| U876_07230 | 0.196 | 4.17E-39 | Down | flagellar biosynthesis protein FlgN |
| U876_07235 | 0.184 | 2.59E-80 | Down | anti-sigma D factor |
| U876_07240 | 0.464 | 3.64E-05 | Down | flagellar basal body P-ring biosynthesis protein FlgA |
| U876_07255 | 0.173 | 3.45E-65 | Down | flagellar basal body rod protein FlgB |
| U876_07260 | 0.183 | 2.23E-33 | Down | flagellar basal body rod protein FlgC |
| U876_07265 | 0.323 | 5.62E-25 | Down | flagellar basal body rod modification protein FlgD |
| U876_07270 | 0.495 | 1.79E-17 | Down | flagellar hook protein FlgE |
| U876_07275 | 0.162 | 7.45E-61 | Down | flagellar basal body rod protein FlgF |
| U876_07280 | 0.197 | 1.57E-31 | Down | flagellar basal body rod protein FlgG |
| U876_07285 | 0.218 | 3.52E-36 | Down | flagellar L-ring protein FlgH |
| U876_07290 | 0.271 | 7.57E-35 | Down | flagellar P-ring protein FlgI |
| U876_07340 | 0.335 | 3.96E-18 | Down | histidine kinase |
| U876_07345 | 0.260 | 2.42E-24 | Down | Fis family transcriptional regulator |
| U876_07385 | 0.404 | 2.14E-30 | Down | malate synthase |
| U876_07460 | 0.354 | 1.92E-33 | Down | peptide ABC transporter ATP-binding protein |
| U876_07465 | 0.436 | 1.29E-21 | Down | ABC transporter permease |
| U876_07470 | 0.409 | 1.30E-33 | Down | amino acid ABC transporter substrate-binding protein |
| U876_07560 | 0.418 | 1.86E-23 | Down | aromatic amino acid aminotransferase |
| U876_07635 | 0.431 | 0.000698 | Down | hypothetical protein |
| U876_07825 | 0.497 | 3.22E-10 | Down | sugar ABC transporter substrate-binding protein |
| U876_07935 | 0.412 | 4.19E-14 | Down | nitrite reductase |
| U876_07940 | 0.371 | 1.71E-19 | Down | nitrite reductase |
| U876_07945 | 0.358 | 1.69E-28 | Down | nitrite reductase |
| U876_08030 | 0.430 | 3.46E-24 | Down | iron transporter FeoB |
| U876_08035 | 0.292 | 6.88E-26 | Down | LuxR family transcriptional regulator |
| U876_08055 | 0.441 | 0.024336 | Down | membrane protein |
| U876_08090 | 0.471 | 3.37E-19 | Down | D-3-phosphoglycerate dehydrogenase |
| U876_08250 | 0.325 | 1.96E-16 | Down | hypothetical protein |
| U876_08255 | 0.340 | 3.28E-40 | Down | diguanylate phosphodiesterase |
| U876_08280 | 0.104 | 5.27E-149 | Down | aromatic amino acid transporter |
| U876_08315 | 0.459 | 2.12E-24 | Down | peptidase S8 |
| U876_08350 | 0.479 | 0.018800 | Down | membrane protein |
| U876_08570 | 0.385 | 6.43E-21 | Down | flagellar protein MotY |
| U876_08710 | 0.483 | 2.10E-26 | Down | peptide ABC transporter substrate-binding protein |
| U876_08800 | 0.255 | 8.85E-07 | Down | hypothetical protein |
| U876_08805 | 0.184 | 5.79E-20 | Down | hypothetical protein |
| U876_08810 | 0.072 | 1.18E-14 | Down | hypothetical protein |
| U876_08815 | 0.237 | 0.001914 | Down | hypothetical protein |
| U876_08820 | 0.311 | 3.85E-05 | Down | hypothetical protein |
| U876_08825 | 0.047 | 0.001988 | Down | hypothetical protein |
| U876_08830 | 0.143 | 8.06E-08 | Down | hypothetical protein |
| U876_08840 | 0.141 | 2.46E-06 | Down | hypothetical protein |
| U876_08845 | 0.378 | 0.000367 | Down | hypothetical protein |
| U876_08850 | 0.408 | 2.00E-09 | Down | recombinase RecT |
| U876_08855 | 0.171 | 5.06E-11 | Down | exodeoxyribonuclease VIII |
| U876_08860 | 0.200 | 1.79E-05 | Down | hypothetical protein |
| U876_08865 | 0.220 | 0.001399 | Down | hypothetical protein |
| U876_08870 | 0.178 | 0.001474 | Down | hypothetical protein |
| U876_08875 | 0.073 | 0.030174 | Down | hypothetical protein |
| U876_08880 | 0.274 | 3.67E-07 | Down | hypothetical protein |
| U876_08890 | 0.120 | 2.79E-10 | Down | hypothetical protein |
| U876_08905 | 0.314 | 0.000695 | Down | regulatory protein |
| U876_08910 | 0.250 | 3.20E-06 | Down | hypothetical protein |
| U876_08915 | 0.297 | 0.013265 | Down | hypothetical protein |
| U876_08950 | 0.197 | 0.000376 | Down | hypothetical protein |
| U876_08955 | 0.196 | 5.26E-06 | Down | Rha family transcriptional regulator |
| U876_08960 | 0.239 | 0.000273 | Down | hypothetical protein |
| U876_08965 | 0.189 | 0.006051 | Down | hypothetical protein |
| U876_08980 | 0.169 | 0.002435 | Down | hypothetical protein |
| U876_09040 | 0.377 | 0.007905 | Down | terminase |
| U876_09045 | 0.404 | 0.000289 | Down | portal protein |
| U876_09055 | 0.336 | 0.001166 | Down | hypothetical protein |
| U876_09060 | 0.271 | 1.02E-06 | Down | virion structural protein |
| U876_09070 | 0.230 | 0.044949 | Down | hypothetical protein |
| U876_09075 | 0.181 | 0.004478 | Down | hypothetical protein |
| U876_09080 | 0.310 | 0.026215 | Down | hypothetical protein |
| U876_09095 | 0.357 | 0.000135 | Down | hypothetical protein |
| U876_09255 | 0.456 | 1.08E-18 | Down | hypothetical protein |
| U876_09315 | 0.370 | 1.21E-13 | Down | peptidase S66 |
| U876_09385 | 0.218 | 2.17E-21 | Down | lactate permease |
| U876_09390 | 0.119 | 2.67E-24 | Down | oxidoreductase |
| U876_09395 | 0.163 | 2.43E-27 | Down | (Fe-S)-binding protein |
| U876_09400 | 0.166 | 4.90E-11 | Down | hypothetical protein |
| U876_09405 | 0.272 | 1.40E-17 | Down | 4Fe-4S ferredoxin |
| U876_09515 | 0.330 | 0.018056 | Down | hypothetical protein |
| U876_09615 | 0.361 | 9.83E-20 | Down | hydrogenase 2 small subunit |
| U876_09620 | 0.254 | 1.72E-37 | Down | hydrogenase 2 protein HybA |
| U876_09625 | 0.199 | 2.34E-61 | Down | hydrogenase 2 b cytochrome subunit |
| U876_09630 | 0.319 | 3.24E-45 | Down | hydrogenase 2 large subunit |
| U876_09635 | 0.334 | 3.59E-32 | Down | hydrogenase 2 maturation endopeptidase |
| U876_09640 | 0.498 | 8.39E-11 | Down | hydrogenase biosynthesis protein HybE |
| U876_09665 | 0.447 | 1.92E-05 | Down | peptide ABC transporter ATP-binding protein |
| U876_09695 | 0.427 | 1.50E-05 | Down | hydrogenase maturation protein HypF |
| U876_09845 | 0.442 | 1.48E-07 | Down | hypothetical protein |
| U876_09860 | 0.167 | 7.31E-09 | Down | enterobactin synthase subunit E |
| U876_09870 | 0.434 | 9.99E-09 | Down | peptide synthetase |
| U876_09875 | 0.278 | 3.84E-08 | Down | 2,3-dihydroxybenzoate-2,3-dehydrogenase |
| U876_09880 | 0.396 | 4.25E-15 | Down | peptide synthetase |
| U876_09885 | 0.337 | 1.81E-09 | Down | peptide synthetase |
| U876_09890 | 0.437 | 0.000390 | Down | ABC transporter substrate-binding protein |
| U876_09895 | 0.424 | 0.035187 | Down | phosphopantetheinyl transferase |
| U876_09915 | 0.490 | 1.21E-07 | Down | TonB-dependent receptor |
| U876_09970 | 0.014 | 0 | Down | phospho-2-dehydro-3-deoxyheptonate aldolase |
| U876_10550 | 0.444 | 4.99E-11 | Down | LysR family transcriptional regulator |
| U876_10985 | 0.481 | 5.97E-10 | Down | hypothetical protein |
| U876_11215 | 0.410 | 1.08E-12 | Down | hypothetical protein |
| U876_11315 | 0.203 | 0.001084 | Down | hypothetical protein |
| U876_11360 | 0.247 | 0.025484 | Down | hypothetical protein |
| U876_11380 | 0.369 | 0.012762 | Down | hypothetical protein |
| U876_11450 | 0.415 | 6.20E-07 | Down | hypothetical protein |
| U876_11870 | 0.420 | 2.25E-32 | Down | methylcitrate synthase |
| U876_11985 | 0.468 | 0.014992 | Down | hypothetical protein |
| U876_12020 | 0.451 | 2.50E-29 | Down | peptidase S41 |
| U876_12025 | 0.440 | 9.18E-23 | Down | cytochrome oxidase |
| U876_12090 | 0.425 | 1.51E-07 | Down | sugar ABC transporter ATP-binding protein |
| U876_12125 | 0.461 | 8.62E-26 | Down | NAD-dependent deacetylase |
| U876_12225 | 0.442 | 6.06E-09 | Down | murein transglycosylase |
| U876_12295 | 0.107 | 1.24E-57 | Down | tyrosine transporter TyrP |
| U876_12595 | 0.444 | 0.001227 | Down | NUDIX hydrolase |
| U876_12770 | 0.422 | 2.13E-18 | Down | sodium:proton antiporter |
| U876_12800 | 3.037 | 1.01E-07 | Down | transporter |
| U876_12850 | 0.478 | 2.41E-14 | Down | glycerol-3-phosphate dehydrogenase |
| U876_12855 | 0.500 | 4.53E-10 | Down | glycerol-3-phosphate dehydrogenase |
| U876_12870 | 0.473 | 2.60E-06 | Down | hypothetical protein |
| U876_12875 | 0.426 | 8.66E-32 | Down | cytochrome C nitrite reductase |
| U876_12920 | 0.324 | 3.06E-59 | Down | cysteine desulfhydrase |
| U876_12965 | 0.377 | 0.048576 | Down | siderophore ferric iron reductase |
| U876_13215 | 0.444 | 0.029972 | Down | L-arabinose transporter ATP-binding protein |
| U876_13250 | 0.466 | 1.66E-12 | Down | alpha-galactosidase |
| U876_13640 | 0.391 | 8.21E-08 | Down | hypothetical protein |
| U876_13795 | 0.490 | 0.010331 | Down | hypothetical protein |
| U876_13820 | 0.186 | 0.042935 | Down | tRNA-Ser |
| U876_14025 | 0.300 | 1.49E-07 | Down | protease |
| U876_14030 | 0.273 | 1.62E-14 | Down | protease |
| U876_14230 | 0.198 | 7.39E-31 | Down | N-acetylneuraminate synthase |
| U876_14245 | 0.296 | 1.74E-13 | Down | hypothetical protein |
| U876_14250 | 0.456 | 0.00016 | Down | hypothetical protein |
| U876_14255 | 0.468 | 4.54E-10 | Down | flagellar protein FliS |
| U876_14260 | 0.326 | 6.47E-16 | Down | flagellar hook protein FliD |
| U876_14265 | 0.246 | 1.99E-41 | Down | flagellin |
| U876_14270 | 0.148 | 1.89E-41 | Down | flagellin |
| U876_14285 | 0.092 | 1.47E-149 | Down | flagellin |
| U876_14290 | 0.132 | 2.73E-41 | Down | flagellin |
| U876_14380 | 0.493 | 4.15E-14 | Down | ABC transporter |
| U876_14470 | 0.457 | 5.13E-12 | Down | maltose transporter permease |
| U876_14540 | 0.039 | 8.2E-300 | Down | chorismate mutase |
| U876_14545 | 0.005 | 0 | Down | phospho-2-dehydro-3-deoxyheptonate aldolase |
| U876_14735 | 0.490 | 0.005833 | Down | membrane protein |
| U876_14770 | 0.310 | 1.61E-07 | Down | hypothetical protein |
| U876_14805 | 0.229 | 7.47E-16 | Down | chemotaxis protein |
| U876_14815 | 0.482 | 9.09E-09 | Down | molybdopterin molybdenumtransferase |
| U876_15005 | 0.495 | 4.67E-08 | Down | endonuclease |
| U876_15045 | 0.394 | 2.06E-05 | Down | hypothetical protein |
| U876_15265 | 0.178 | 3.4E-106 | Down | hemolysin |
| U876_15415 | 0.435 | 1.27E-10 | Down | C4-dicarboxylate transporter |
| U876_15680 | 0.484 | 0.017490 | Down | hypothetical protein |
| U876_15700 | 0.283 | 9.18E-06 | Down | head protein |
| U876_15705 | 0.212 | 0.000353 | Down | hypothetical protein |
| U876_15765 | 0.099 | 0.008763 | Down | secretion activator protein |
| U876_15805 | 0.395 | 0.000242 | Down | sulfate transporter |
| U876_15825 | 0.350 | 0.000350 | Down | hypothetical protein |
| U876_15830 | 0.356 | 0.003164 | Down | DNA transposition protein |
| U876_15835 | 0.355 | 2.90E-08 | Down | integrase |
| U876_15840 | 0.304 | 0.005465 | Down | hypothetical protein |
| U876_15870 | 0.498 | 2.45E-09 | Down | hypothetical protein |
| U876_16110 | 0.461 | 1.30E-09 | Down | cytochrome C biogenesis protein CcmE |
| U876_16150 | 0.480 | 6.18E-06 | Down | cobalamin biosynthesis protein CobQ |
| U876_16170 | 0.479 | 1.47E-24 | Down | chemotaxis protein CheA |
| U876_16195 | 0.375 | 2.17E-32 | Down | flagellar biosynthesis regulator FlhF |
| U876_16200 | 0.487 | 3.29E-19 | Down | flagellar biosynthesis protein FlhA |
| U876_16245 | 0.424 | 3.05E-15 | Down | flagellar hook-length control protein FliK |
| U876_16250 | 0.299 | 2.50E-09 | Down | flagellar export protein FliJ |
| U876_16275 | 0.201 | 6.57E-20 | Down | flagellar hook-basal body protein FliE |
| U876_16310 | 0.218 | 0.026330 | Down | peptide chain release factor |
| U876_16315 | 0.460 | 0.000101 | Down | peptidase C39 |
| U876_16400 | 0.385 | 4.16E-11 | Down | TetR family transcriptional regulator |
| U876_16435 | 0.343 | 0.024948 | Down | ethanolamine utilization protein EutN |
| U876_16600 | 0.333 | 0.037368 | Down | tRNA-Ser |
| U876_16825 | 0.348 | 3.19E-13 | Down | ornithine decarboxylase |
| U876_16935 | 0.267 | 0.002934 | Down | AraC family transcriptional regulator |
| U876_17085 | 0.497 | 2.94E-07 | Down | alpha-amylase |
| U876_17090 | 0.380 | 1.89E-15 | Down | glutathione S-transferase |
| U876_17120 | 0.330 | 6.95E-11 | Down | diguanylate cyclase |
| U876_17320 | 0.487 | 3.89E-26 | Down | maltoporin |
| U876_17375 | 0.463 | 2.80E-19 | Down | hypothetical protein |
| U876_17695 | 0.069 | 2.81E-17 | Down | hypothetical protein |
| U876_17700 | 0.081 | 2.04E-28 | Down | hypothetical protein |
| U876_17705 | 0.277 | 1.06E-14 | Down | hypothetical protein |
| U876_17915 | 0.233 | 0.000162 | Down | transposase |
| U876_18000 | 0.431 | 2.80E-20 | Down | 7-cyano-7-deazaguanine synthase |
| U876_18110 | 0.493 | 2.28E-05 | Down | ABC transporter substrate-binding protein |
| U876_18555 | 0.245 | 5.79E-07 | Down | ligand-gated channel protein |
| U876_18565 | 0.429 | 1.32E-11 | Down | isochorismatase |
| U876_18840 | 0.329 | 9.99E-07 | Down | hypothetical protein |
| U876_18845 | 0.402 | 1.56E-15 | Down | hypothetical protein |
| U876_18905 | 0.406 | 0.001598 | Down | hypothetical protein |
| U876_19285 | 0.341 | 3.25E-18 | Down | 2-isopropylmalate synthase |
| U876_19290 | 0.474 | 2.70E-07 | Down | 3-isopropylmalate dehydrogenase |
| U876_19615 | 0.014 | 1.61E-13 | Down | nucleoside triphosphate hydrolase |
| U876_19620 | 0.209 | 6.52E-64 | Down | (p)ppGpp synthetase |
| U876_19685 | 0.374 | 0.031614 | Down | tagatose-bisphosphate aldolase |
| U876_19750 | 0.470 | 0.000117 | Down | hypothetical protein |
| U876_19770 | 0.496 | 0.000255 | Down | membrane protein |
| U876_19785 | 0.407 | 2.18E-11 | Down | thiol:disulfide interchange protein DsbD |
| U876_19875 | 0.453 | 1.11E-18 | Down | prolyl endopeptidase |
| U876_20060 | 0.224 | 2.83E-28 | Down | MarR family transcriptional regulator |
| U876_20140 | 0.458 | 0.004653 | Down | hypothetical protein |
| U876_20480 | 0.310 | 1.81E-06 | Down | iron ABC transporter permease |
| U876_20515 | 0.390 | 1.99E-09 | Down | glutathione peroxidase |
| U876_20665 | 0.339 | 8.89E-29 | Down | hypothetical protein |
| U876_20730 | 0.140 | 0.049772 | Down | sulfate transporter subunit |
| U876_20780 | 0.470 | 1.04E-15 | Down | acetylglutamate kinase |
| U876_21140 | 0.119 | 6.4E-113 | Down | fimbrial protein |
| U876_21145 | 0.132 | 7.73E-28 | Down | fimbrial protein |
| U876_21150 | 0.119 | 2.0E-134 | Down | fimbrial protein |
| U876_21155 | 0.091 | 3.6E-114 | Down | fimbrial assembly protein |
| U876_21160 | 0.032 | 6.1E-116 | Down | hypothetical protein |
| U876_21165 | 0.119 | 6.95E-37 | Down | fimbrial protein |
| U876_21185 | 0.421 | 1.99E-07 | Down | superoxide dismutase |
| U876_21190 | 0.393 | 4.63E-05 | Down | hypothetical protein |
| U876_21245 | 0.275 | 8.93E-06 | Down | hypothetical protein |
| U876_21330 | 0.437 | 8.82E-05 | Down | multidrug ABC transporter permease |
| U876_21565 | 0.345 | 3.91E-06 | Down | thiamine biosynthesis protein ThiC |
| U876_21570 | 0.373 | 2.32E-35 | Down | aerolysin |
| U876_21685 | 0.180 | 6.13E-73 | Down | fimbrial protein |
| U876_21775 | 0.484 | 3.47E-09 | Down | MFS transporter |
| U876_21960 | 0.497 | 0.000554 | Down | calcium:proton antiporter |
| U876_22130 | 0.410 | 1.40E-34 | Down | C4-dicarboxylate ABC transporter substrate-binding protein |
| U876_22135 | 0.491 | 1.04E-15 | Down | protease 2 |
| U876_22185 | 0.262 | 7.89E-07 | Down | tRNA-Asp |
| U876_22580 | 0.475 | 5.18E-26 | Down | hypothetical protein |
| U876_22815 | 0.469 | 1.50E-17 | Down | SAM-dependent methyltransferase |
| U876_22860 | 0.061 | 0.013382 | Down | tRNA-Thr |
| U876_23040 | 0.438 | 3.32E-05 | Down | hypothetical protein |
| U876_23100 | 0.489 | 4.25E-21 | Down | ketol-acid reductoisomerase |
| U876_23250 | 0.132 | 2.69E-47 | Down | hypothetical protein |
| U876_23305 | 0.463 | 0.001456 | Down | 6-phospho-alpha-glucosidase |
| U876_23320 | 0.352 | 7.19E-15 | Down | branched-chain amino acid transporter permease subunit LivH |
| U876_23325 | 0.405 | 2.07E-20 | Down | leucine/isoleucine/valine transporter permease subunit |
| U876_23565 | 0.455 | 2.41E-14 | Down | hypothetical protein |
| U876_23580 | 0.262 | 1.80E-07 | Down | carbon-phosphorus lyase complex subunit PhnJ |
| U876_23585 | 0.357 | 1.63E-15 | Down | hypothetical protein |
| U876_23590 | 0.458 | 9.98E-08 | Down | phosphonate C-P lyase system protein PhnH |
| U876_23595 | 0.369 | 2.61E-20 | Down | phosphonate C-P lyase system protein PhnG |
| U876_23600 | 0.378 | 7.41E-35 | Down | hypothetical protein |
| U876_23605 | 0.310 | 6.12E-18 | Down | ABC transporter ATP-binding protein |
| U876_23610 | 0.207 | 2.20E-33 | Down | ABC transporter permease |
| U876_23615 | 0.285 | 7.01E-32 | Down | hypothetical protein |
| U876_23620 | 0.437 | 0.002011 | Down | hypothetical protein |
| U876_23795 | 0.482 | 1.15E-16 | Down | cobalamin biosynthesis protein CobQ |
| U876_23855 | 0.415 | 1.39E-06 | Down | hypothetical protein |
| U876_24240 | 0.455 | 2.41E-14 | Down | hypothetical protein |
| U876_24255 | 0.262 | 1.80E-07 | Down | carbon-phosphorus lyase complex subunit PhnJ |
| U876_24260 | 0.357 | 1.63E-15 | Down | hypothetical protein |
| U876_24265 | 0.458 | 9.98E-08 | Down | phosphonate C-P lyase system protein PhnH |
| U876_24270 | 0.369 | 2.61E-20 | Down | phosphonate C-P lyase system protein PhnG |
| U876_24275 | 0.378 | 7.41E-35 | Down | hypothetical protein |
| U876_24280 | 0.310 | 6.12E-18 | Down | ABC transporter ATP-binding protein |
| U876_24285 | 0.207 | 2.20E-33 | Down | ABC transporter permease |
| U876_24290 | 0.285 | 7.01E-32 | Down | hypothetical protein |
| U876_24295 | 0.437 | 0.002011 | Down | hypothetical protein |
